# Cellular characterisation of advanced osteoarthritis knee synovium

**DOI:** 10.1186/s13075-023-03110-x

**Published:** 2023-08-23

**Authors:** Jolet Y. Mimpen, Robert Hedley, Anna Ridley, Mathew J. Baldwin, Dylan Windell, Ananya Bhalla, Lorenzo Ramos-Mucci, Christopher D. Buckley, Mark C. Coles, Abtin Alvand, Andrew J. Price, Andrew J. Carr, Stephanie G. Dakin, Sarah J. B. Snelling

**Affiliations:** 1 Botnar Research Centre, Nuffield Department of Orthopaedics Rheumatology and Musculoskeletal Sciences, University of Oxford, Oxford, UK; 2 Kennedy Institute of Rheumatology, Nuffield Department of Orthopaedics Rheumatology and Musculoskeletal Sciences, University of Oxford, Oxford, UK

**Keywords:** Osteoarthritis, Synovium, Fibroblasts, Macrophages, T cells, B cells, Flow cytometry, Immunofluorescence, CCR6

## Abstract

**Objectives:**

Osteoarthritis (OA) is increasingly recognised as a whole joint disease, with an important role for synovium. However, the repertoire of immune cells and fibroblasts that constitute OA synovium remains understudied. This study aims to characterise the cellular composition of advanced OA synovium and to explore potential correlations between different cell types and patient demographics or clinical scores.

**Methods:**

Synovium, collected from 10 patients with advanced OA during total knee replacement surgery, was collagenase-digested, and cells were stained for flow cytometry analysis. Formalin-fixed paraffin-embedded synovium was sectioned, stained with immunofluorescence, and imaged using the multiplex Cell DIVE platform. Patient demographics and clinical scores were also collected.

**Results:**

The proportion of immune cells in OA synovium varied between patients (8–38% of all cells). Macrophages and T cells were the dominant immune cell populations, together representing 76% of immune cells. Age positively correlated with the proportion of macrophages, and negatively correlated with T cells. CCR6+ T cells were found in 6/10 patients; these patients had a higher mean Kellgren-Lawrence grade across the three knee compartments. Immunofluorescence staining showed that macrophages were present in the lining as well as distributed throughout the sublining, while T and B cells were mainly localised near vessels in the sublining. Fibroblast subsets (CD45−PDPN+) based on the expression of CD34/CD90 or FAP/CD90 were identified in all patient samples, and some populations correlate with the percentage of immune cells or clinical scores. Immunofluorescence staining showed that FAP expression was particularly strong in the lining layer, but also present throughout the sublining layer. CD90 expression was exclusively found around vessels in the sublining, while CD34 was mostly found in the sublining but also occasionally in the lining layer.

**Conclusions:**

There are significant differences in the relative proportions and subsets of immune cells in OA synovium; exploratory correlative analyses suggest that these differences might be correlated with age, clinical scores, or fibroblast subsets. Additional studies are required to understand how different cell types affect OA pathobiology, and if the presence or proportion of cell subsets relates to disease phenotypes.

**Supplementary Information:**

The online version contains supplementary material available at 10.1186/s13075-023-03110-x.

## Background

In recent years the pathophysiology of osteoarthritis (OA) has shifted from defining OA as a degenerative “wear-and-tear” disease of articular cartilage to a disease involving all joint tissues. This further supports evidence of OA as a multifactorial disease with a complex pathophysiology [1, 2]. Although historically referred to as “non-inflammatory” arthritis, many patients with OA report symptoms related to joint inflammation, including morning stiffness, warmth, pain, and joint effusion [3]. These symptoms can be attributed to synovial thickening and/or synovial fluid effusion, pointing to synovial inflammation (synovitis) as an associated pathological feature [4].

Synovium consists of a fibrous sub-lining layer and a thin cellular lining layer [5]. In OA patients synovium is characterised by synovial lining hyperplasia, fibrosis of the synovial sub-lining, and increased vascularisation [6]. The increase in synovial vascularisation is thought to facilitate the influx of leukocytes in response to factors secreted in the OA joint [7]. The predominant immune cell types in OA synovium are reported to be T cells and macrophages. Although an outdated classification, both classically activated “M1” and alternatively activated “M2” macrophages have previously been found in OA synovium. However, studies are contradictory regarding the relative proportions of each subtype [8–10]. More recent studies using healthy and rheumatoid arthritis (RA) synovium have proposed new nomenclature for different synovial macrophage subsets, including CX_3_CR1+/TREM2+ lining macrophages and subsets based on the expression of CD163, CD206, and MERTK [11, 12]. Small numbers of other immune cells have also been found in OA synovium, including B cells, natural killer cells, dendritic cells, mast cells, monocytes, neutrophils, and plasma cells [3, 7, 13]. It is currently unclear if and how different immune cell populations relate to each other as specific types of immune cells are frequently studied in isolation.

Fibroblasts are the most common cell type in connective tissues, including the synovium. In healthy synovium, synovial fibroblasts maintain the volume and composition of synovial fluid and produce matrix components that provide strength and structure to synovium [14, 15]. However, synovial fibroblasts can also contribute to inflammation, fibrosis, and tissue destruction [16]. There is active cross-talk between immune cells and fibroblasts [17], and fibroblasts can undergo phenotypic changes as a consequence of inflammation [18]. CD34, CD44, CD55, CD90 (also called THY1), cadherin 11, VCAM1 (CD34), CD248, fibroblast activation protein (FAP), and podoplanin (PDPN) are highly expressed on fibroblasts in inflammatory conditions, [19–28], and have all been used to distinguish different fibroblast subsets in rheumatoid arthritis (RA). Mizoguchi et al. describe three functionally distinct disease-associated fibroblasts subsets in synovium based on expression of CD34 and CD90 [22]. Croft et al. identified two distinct fibroblasts subsets that drive inflammation (FAP+CD90+ sublining cells) and tissue damage (FAP+CD90− lining cells) in (models of) RA using single-cell transcriptional analysis. Cytometry time of flight (CyTOF) mass spectrometry analysis showed that FAP+CD90+ cells were increased in RA synovium compared to OA, and in RA these cells positively correlated with markers of systemic and tissue inflammation [21]. However, it remains unclear if there is any relationship between these fibroblast populations, immune cell populations, and clinical scores in OA synovium.

While synovitis is increasingly recognised as an important pathological feature of OA, the immune cells and (pathogenic) fibroblasts in OA synovium remain understudied. Improved understanding of the pathophysiology of synovitis in OA has the potential to enable the development and testing of treatments for patients, especially OA patients with symptoms related to joint inflammation. Therefore, the overarching aim of this study was to characterise the cellular subsets in advanced OA synovium using flow cytometry and multiplex imaging and to study the relationship between different cellular subsets and clinical scores.

## Methods

### Ethical approval

Ethical approval for the Oxford Musculoskeletal Biobank (09/H0606/11 and 19/SC/0134) was granted by the Oxford Research Ethics Committee B for all work on human synovium, and written informed consent according to the Declaration of Helsinki was obtained from all patients.

### Patients and clinical details

Synovium was collected from advanced knee OA patients during routine total knee replacement surgery. Patients were recruited sequentially. Patients with inflammatory arthritis or secondary OA due to trauma were excluded. The age, gender, affected knee, and body mass index (BMI) were collected (Table 1). Kellgren-Lawrence (KL) grade for each knee compartment (medial, lateral, patellofemoral) was scored by one blinded clinician (MJB) based on X-rays (AP and lateral) according to the criteria as described by Kellgren & Lawrence [29]. The mean compartmental KL grade of the three knee compartments was used in this study to illustrate the global disease burden across the joint. Results using the classic KL grading representing the highest radiographic severity either using only the medial and lateral compartments (m/l only) or all three knee compartments (m/l/pf) can be found in the Additional Files. Pre-operative Oxford Knee Score (OKS) [30] was obtained from the Electronic Patient Record if available. Due to the high number of missing values (4 out of 10), OKS was not used in any of the exploratory correlative analyses. Tissue from the first 10 patients was used for flow cytometry analysis; additional samples were collected for imaging.

**Table 1:** Patient demographics and clinical characteristics

**Patient no.**	**Gender**	**Age**	**Affected knee**	**BMI**	**Mean compartmental KL grade (m, l, pf)**	**OKS**
**1**	Female	64	Right	34.8	2.67 (4, 2, 2)	*Not available*
**2**	Female	58	Left	33.9	2 (0, 2, 4)	*Not available*
**3**	Female	80	Left	34.0	2.33 (4, 3, 0)	25
**4**	Male	62	Right	40.8	4 (4, 4, 4)	*Not available*
**5**	Female	72	Left	40.2	2.33 (3, 2, 2)	20
**6**	Female	61	Right	24.5	1.33 (2, 1, 1)	16
**7**	Female	71	Right	28.5	3.33 (4, 3, 3)	*Not available*
**8**	Male	75	Right	37.9	3.33 (3, 3, 4)	31
**9**	Female	68	Right	28.5	4 (4, 4, 4)	26
**10**	Female	77	Left	33.9	2 (0, 2, 4)	21
**Mean (range)**	20% male/ 80% female	68.8 (58–80)	40% left/ 60% right	33.7 (24.5–40.8)	2.73 (1.33–4)	23.2 (16–31)

*BMI* Body mass index, *KL grade* Kellgren-Lawrence grade, *m, l, pf* medial, lateral, and patellofemoral compartment. KL grade is reported as the mean of the KL grades of the three knee compartments (medial, lateral, and patellofemoral) to demonstrate the global disease burden across the joint

### Flow cytometry analysis

#### Tissue collection and digestion

Fresh OA synovium was collected in R10 media (RPMI (Gibco, Fisher Scientific, Loughborough, UK) with 10% Foetal Bovine Serum (FBS) (Labtech International, Heathfield, UK) and 1% Penicillin/Streptomycin (P/S) (Gibco)). Tissue was washed in media and fat was trimmed. Tissue was placed in fresh R10 media and left overnight at 37 °C. The next morning, the tissue was mechanically homogenised and digested in digestion media (10 mL of 5 mg/ml filtered type II collagenase (Worthington Biochemical Corporation, Thermo Fisher Scientific, Waltham, MA, USA) in R0 media (serum-free RPMI with 1% P/S) for each cm^3^ of tissue) for 2 h at 37 °C with gentle agitation. Cold R10 media was added to stop the reaction, the mixture was then filtered using a 70-µm cell strainer, centrifuged, and reconstituted in R10 media. Brefeldin and Monensin (both BioLegend, London, UK; 1:1000) were added to each sample, vortexed briefly, and incubated at 37 °C for 4 h.

#### Extracellular staining and fixation

After incubation, cells were washed twice with Cell Staining Buffer (CSB) (BioLegend), and incubated in Fc-blocker (BioLegend) for 10 min at 4 °C in the dark. A mixture of extracellular antibodies or corresponding isotype controls with live/dead staining in CSB (Additional Files 1 and 2) was added and incubated for 20 min in the dark on ice. After incubation, cells were washed twice, and incubated in Fixation Buffer (BioLegend) for 20 min in the dark at room temperature (RT). After fixing, cells were washed in CSB and left in the dark at 4 °C until intracellular staining.

#### Permeabilization and intracellular staining

Cells were incubated in permeabilization buffer (Invitrogen) for 30 min at 4 °C. After incubation, cells were spun down and incubated in a mixture of intracellular antibodies or corresponding isotype controls (Additional File 2) in permeabilization buffer for 60 min at 4 °C in the dark. Cells were washed twice with permeabilization buffer before CSB was added for analysis.

#### Preparing compensation controls

For compensation controls, OneComp eBeads compensation beads (Invitrogen) were stained on ice, washed, fixed in Fixation Buffer, washed again, and resuspended in CSB for analysis.

#### Flow cytometry runs and analysis

Single-cell suspensions were interrogated using a LSR Fortessa Cell Analyzer flow cytometer (BD Biosciences) with FACS Diva software 8.0.1 (BD Biosciences). Daily baseline performance checks were performed using Cytometry Setup and Tracking Research Beads (BD Biosciences). FlowJo software 10.6.1 (BD Biosciences) was used for analysis. Compensation beads were first used to set correct photomultiplier tubes detectors voltages, then to calculate the amount of fluorescent spill-over to subtract from each channel. Viable cells were selected by separating cells from debris (SSC-A vs FSC-A), gating on single cells (SSC-H vs SSC-W), and finally selecting cells negative for the live/dead viability dye (viability dye vs SSC-A) (Additional File 3).

### Immunofluorescence staining and imaging using Cell DIVE

Synovial samples were immersed in 10% buffered formalin for 0.5 mm/h, embedded in paraffin wax before cutting 5 mm tissue sections and baking onto adhesive glass slides. All protocols were performed in accordance with the Cell DIVE Platform (GE Research, Niskayuna, NY, USA). Tissue sections were baked overnight at 60 °C prior to slide clearing. Slides were deparaffinised in xylene followed by rehydration with 100%, 95%, 70%, 50% ethanol, and PBS; each step was performed twice for 5 min. Tissue slides were permeabilised in PBS with 0.3% Triton X-100 for 10 min and washed with PBS for 5 min. Antigen retrieval was performed using the NxGen decloaking chamber (Biocare Medical, Pacheco, CA, USA) using two antigen retrieval solutions (Citrate and Tris-based solutions, pH 6 and 9, respectively). Slides were blocked overnight at 4°C in PBS with 3% BSA and 10% donkey serum (Bio-Rad), stained with DAPI (ThermoFisher) and mounted using mounting media (4% propyl gallate, 50% glycerol; Sigma-Aldrich). An initial scanplan was acquired at 10X magnification to select regions of interest followed by a background imaging round at 20X to acquire background autofluorescence, which was subtracted from the following staining round. Slides were decoverslipped in PBS; an antibody mixture (unconjugated primary antibodies for the first round of staining and conjugated antibodies for subsequent staining) was prepared and incubated for 1 h at RT or overnight at 4 °C using HybriSlip covers (Sigma-Aldrich). Slides were washed thrice in PBS for 5 min with gentle agitation. An antibody mixture with appropriate secondary antibodies was prepared and slides were incubated for 1 h at RT, washed thrice with PBS, re-coverslipped, and imaged. After imaging, a bleaching round was performed by decoverslipping and incubating the tissue slides twice for 15 min in 0.5M NaHCO_3_ (pH 11.2) and 3% H_2_O_2_ with a 1-min wash in between, followed by 3 washes in PBS and a 2-min DAPI recharge. Slides were then re-coverslipped and a bleached image was acquired which was subtracted from the following staining round. Staining and bleaching rounds were repeated until completion of 5 staining rounds (All antibodies are listed in Additional File 4).

### Statistical analysis

Statistical analyses were performed using GraphPad Prism 8.1.2 (GraphPad Software, La Jolla, CA, USA). Two-way ANOVA with Sidak’s multiple comparison tests were used to test for differences in cell subsets and Mann-Whitney tests were done to test for differences in age, BMI, and mean compartmental KL grade between CCR6+ versus CCR6− groups and T cell dominant versus macrophage dominant groups. Correlation tests using Pearson’s correlation coefficient were used for all correlations tested with two continuous variables. If one of the variables was on an ordinal scale, a non-parametric Spearman correlation was used. For the trend lines, data sets were tested for a normal or log-normal distribution using the Anderson-Darling, D’Agostino & Pearson, Shapiro-Wilk, and Kolmogorov-Smirnov tests and results used to model a linear, a semi-log or log-log line. A trend was noted when *p*-value<0.1; differences were considered significant when *p*-value<0.05. Data is shown as mean ± SD unless otherwise stated.

## Results

### The proportion of immune cells is highly variable in advanced OA synovium

The cellular composition of synovium from 10 sequentially recruited advanced OA patients undergoing total knee replacement (from now on referred to as “advanced OA patients”) was studied using a variety of immune and stromal cell markers with flow cytometry. Patient demographics and clinical demographics are described in Table 1. Oxford Knee Score was only available for 6 patients, which is why it was not used for further analysis. KL grade scoring system was used to score each of the three joint compartments (medial, lateral, and patellofemoral) and the mean of these three scores is reported as the mean compartmental KL grade to demonstrate the global disease burden across the joint.

**Fig. 1:**
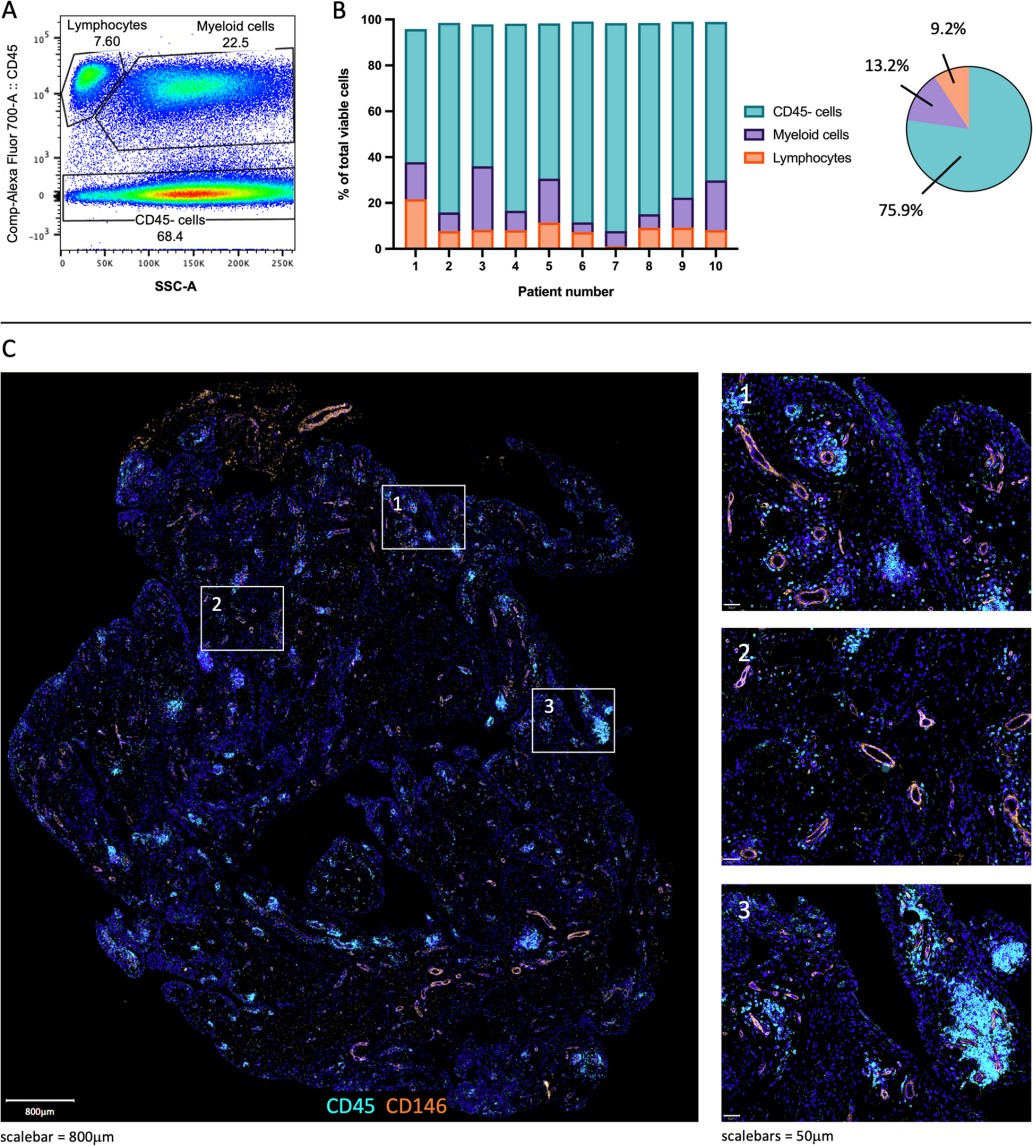
CD45 cell subsets in advanced OA synovium. **A** Representative example of flow cytometry plot gating lymphocytes, myeloid cells, and CD45− cells using SSC-A vs CD45. **B** Relative frequencies (%) of lymphocyte, myeloid, and CD45− subsets as a representation of all viable cells. *n*=10. **C** Representative immunofluorescence imaging of immune cells (CD45, cyan) in advanced OA synovium; DAPI staining (blue) was used to visualise nuclei and CD146 (orange) to visualise vessels

CD45 was used to separate all viable cells into three categories: leukocytes, myeloid cells, and CD45− cells, mainly containing fibroblasts (Fig. 1A, B, Additional File 5). The percentage of immune (CD45+) cells differed between patients (7.7–37.8%), especially in the proportion of myeloid cells. While there is a similar proportion of leukocytes for 8/10 patients, there is a large variation in the proportion of myeloid cells (4.3–27.8%). Multimodal imaging using advanced OA knee synovium was used to spatially locate the identified cell populations. Immune cells were mainly present in clusters around blood vessels, but also in the lining layer and scattered in the sublining layer (Fig. 1C).

### T cells and macrophages are the predominant immune cells in advanced OA synovium

**Fig. 2:**
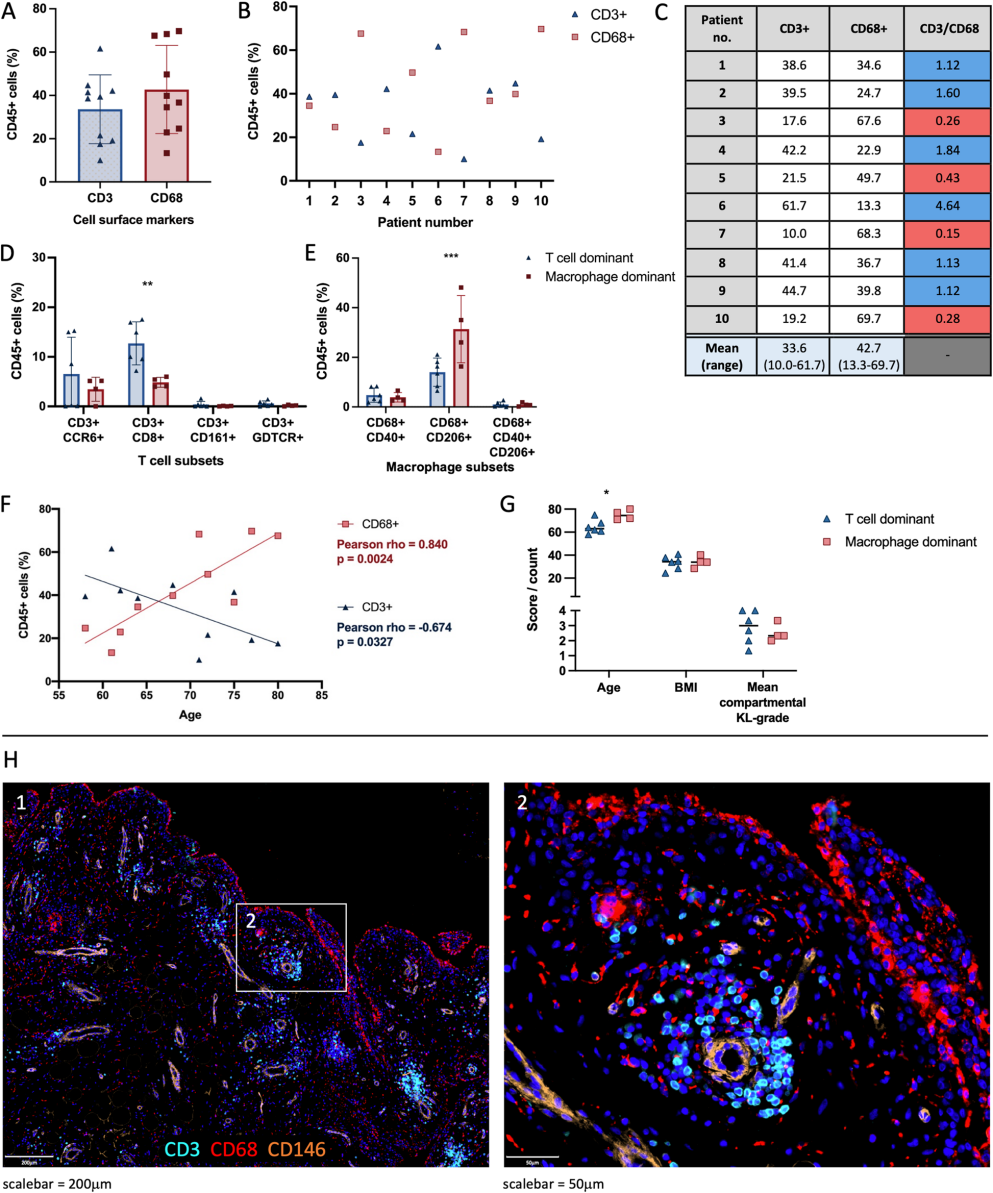
The relative frequency (%) and localisation of macrophages (CD68+) and T cells (CD3+) cells in advanced OA synovium as a percentage of immune cells (CD45+). **A**–**C** Flow cytometry was used to reveal the relative frequencies (%) of macrophages and T cells. **D**–**E** The differences in relative frequency (%) of T cell subsets (**D**) and macrophage subsets (**E**) between T cell dominant (blue) or macrophage dominant (red) patients. **F** Relationship between the relative frequency (%) of macrophages and T cells and the age of the patient. **G** The differences in age, BMI, and mean compartmental Kellgren-Lawrence (KL) grade between T cell dominant (blue) or macrophage dominant (red) patients. *n*=10. **H** Representative immunofluorescence staining of T cells (CD3, cyan) and macrophages (CD68, red); DAPI staining (blue) was used to visualise nuclei and CD146 (orange) to visualise vessels

We confirmed by flow cytometry that macrophages (CD68+) and T cells (CD3+) represent the majority of all immune cell types (CD45+) in advanced OA synovium (43% and 33%, respectively; Fig. 2A–C). Six patients had a higher proportion of T cells than macrophages (1.12–4.64-fold increase); in these patients, there was a significant increase in CD8+ T cells (p=0.0061), but not CCR6+, CD161+ or GDTCR+ T cells (Fig. 2D). Four patients had a higher proportion of macrophages than T cells, which seems to be driven by a significant increase in CD206+ macrophages (*p*=0.0002), but not CD40+ or CD40+CD206+ macrophages (Fig. 2E). Exploratory correlative analyses between cell subsets and patient demographics and clinical scores were carried out. T cells and macrophages as a percentage of all immune cells showed a significant negative (rho=−0.674, *p*=0.033) and positive correlation (rho=0.84, p=0.0024), respectively, with the age of the patient (Fig, 2F). No correlation was found with BMI or mean compartmental KL grade (Fig. 2G, Additional File 6). Immunofluorescence staining showed that T cells were mainly located near vessels; macrophages were found in the lining and scattered around the sublining layer (Fig. 2D).

### Presence of CCR6+ T cells might indicate a subgroup of patients

**Fig. 3:**
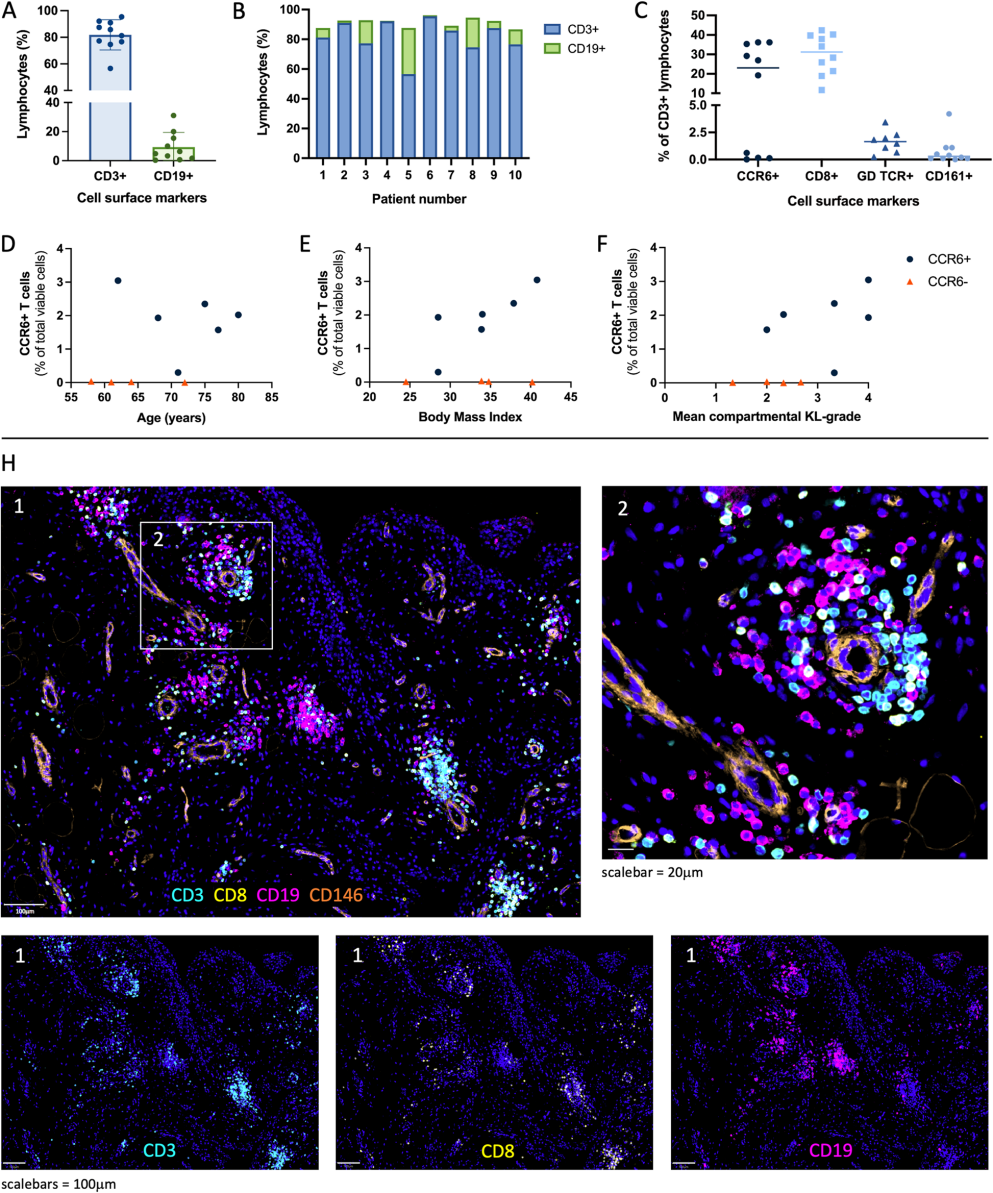
Lymphocytes in advanced OA synovium. **A**,**B** Relative frequency (%) of T cells (CD3+) and B cells (CD19+) as a percentage of lymphocytes. **C** Relative frequency of T cell subsets, including CCR6+, CD8+, GDTCR+, and CD161+ T cells. **D**-**G** Relationship between the relative frequency (%) of CCR6+ T cells and age in years (**D**), body mass index (**E**), and mean compartmental Kellgren-Lawrence (KL) grade (**F**). n=10 (**H**) Representative immunofluorescence staining of CD3 (cyan), CD8 (yellow), CD19 (pink) in advanced OA synovium; DAPI staining (blue) was used to visualise nuclei and CD146 (orange) to visualise vessels

Flow cytometry analysis confirmed the presence of both B cells (CD19+) and T cells (CD3+) in the leukocyte population of each patient. The proportion of B cells was highly variable between patients, with percentages ranging from 0.3 to 31.3% of all leukocytes (Fig. 3A–B, Additional File 7). T cells were further characterised using the surface markers CCR6, CD8, CD161, and GDTCR (Fig. 3C). T cells expressing CCR6, a protein often found on T helper type 17 (Th17) cells and regulatory T (Treg) cells, were only found in 6 of the 10 patients (19–36% of T cells). Upon further analysis, we found that patients with or without CCR6+ T cells were not significantly different in age (*p*=0.1143) or BMI (*p*=0.9429), but patients with CCR6+ T cells tended to have a higher mean compartmental KL grade (higher degree of joint damage, *p*=0.1000) (Fig. 3D–F). This suggests that CD3+CCR6+ cell status might represent a subgroup of patients.

Immunofluorescence staining revealed that both T and B cells were mostly clustered together near vessels in the sublining (Fig. 3H). A large portion of T cells expressed CD8, commonly associated with cytotoxic T cells.

### CD68+CD206+ macrophages are the predominant myeloid cell subset

**Fig. 4:**
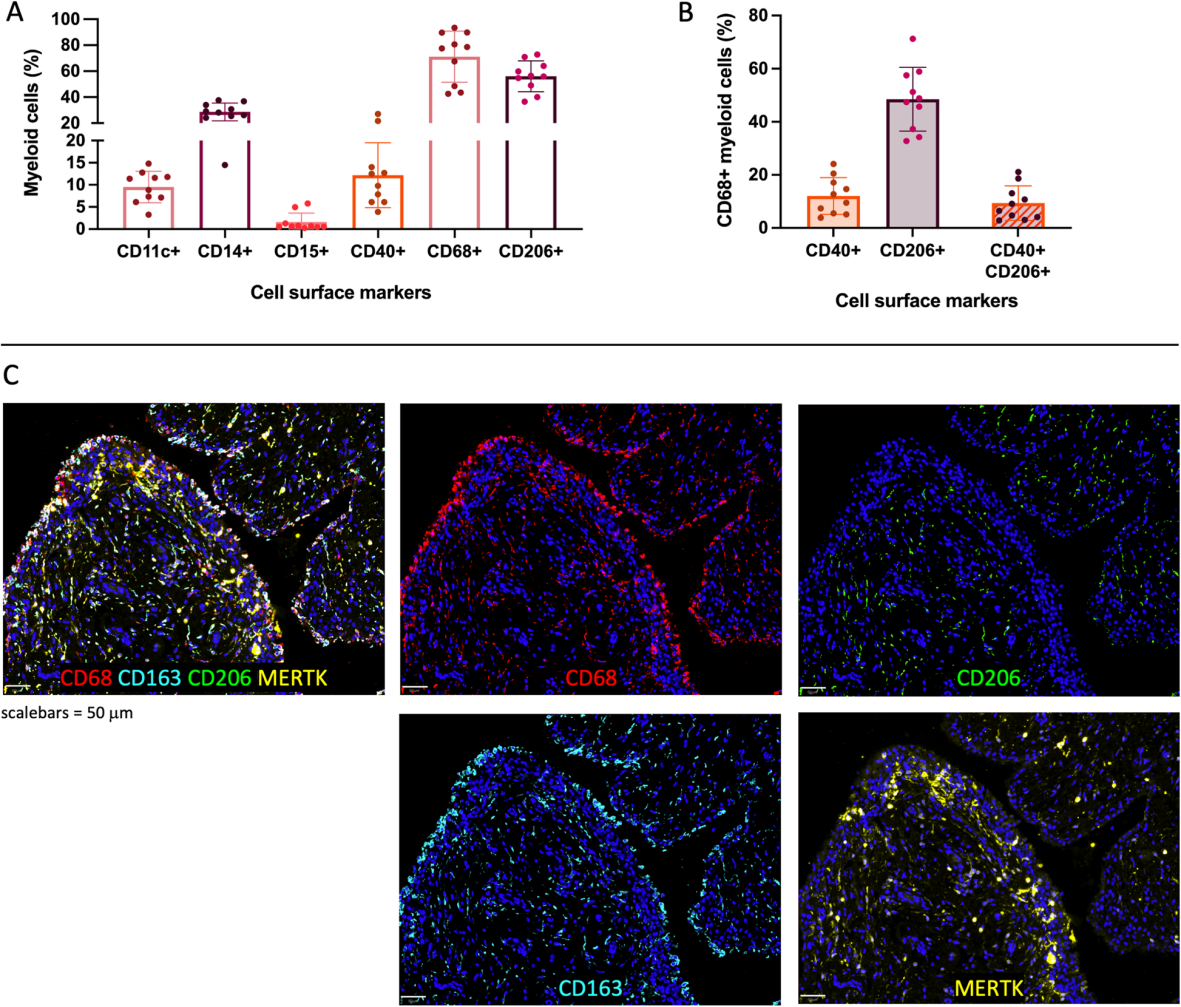
Myeloid cells in advanced OA synovium. **A** Relative frequency (%) of myeloid cells positive for CD11c, CD14, CD15, CD40, CD68, or CD206. **B** Relative frequency (%) CD40 and/or CD206 positive macrophage populations. **C** Immunofluorescence staining of macrophage markers CD68 (red), CD206 (green), CD163 (cyan), and MERTK (yellow) in advanced OA synovium; DAPI staining (blue) was used to visualise nuclei

Flow cytometry analysis revealed that the majority (71%) of myeloid cells expressed the macrophage marker CD68. After CD68, the most prominently expressed cell surface markers were CD206 (56%), CD14 (29%), CD40 (12.2%), CD11c (9.5%), and CD15 (1.6%) (Fig. 4A, Additional File 8). Of the macrophages, approximately half were positive for CD206, 12% were positive for CD40, and 9.4% were positive for both CD40 and CD206 (Fig. 4B).

Immunofluorescence staining showed that macrophages can be found across both the lining and sublining layers (Fig. 4C). CD206 expression was largely absent from the lining layer, while its expression was found throughout the sublining. Expression of macrophage markers CD163 and MERTK was also studied using immunofluorescence. CD163 was found both on the outside of the lining as well as scattered throughout the sublining, while expression of MERTK was located towards the inside of the lining layer as well as some parts of the sublining. The expression of CD206 in the sublining is largely co-localised with CD163. While CD163 and MERTK co-localised in some areas, in most areas only one of the two markers was expressed.

### The majority of OA fibroblasts have an immune effector phenotype

Flow cytometry analysis was used to study the CD45− population (Fig. 1), mainly comprising fibroblasts, for the expression of CD34 and the fibroblast activation markers CD90, FAP, and PDPN. FAP and PDPN were expressed on 78% and 77% of CD45− cells, respectively, while expression of CD90 (59.5%) and CD34 (47.6%) were relatively lower (Additional File 9).

**Fig. 5:**
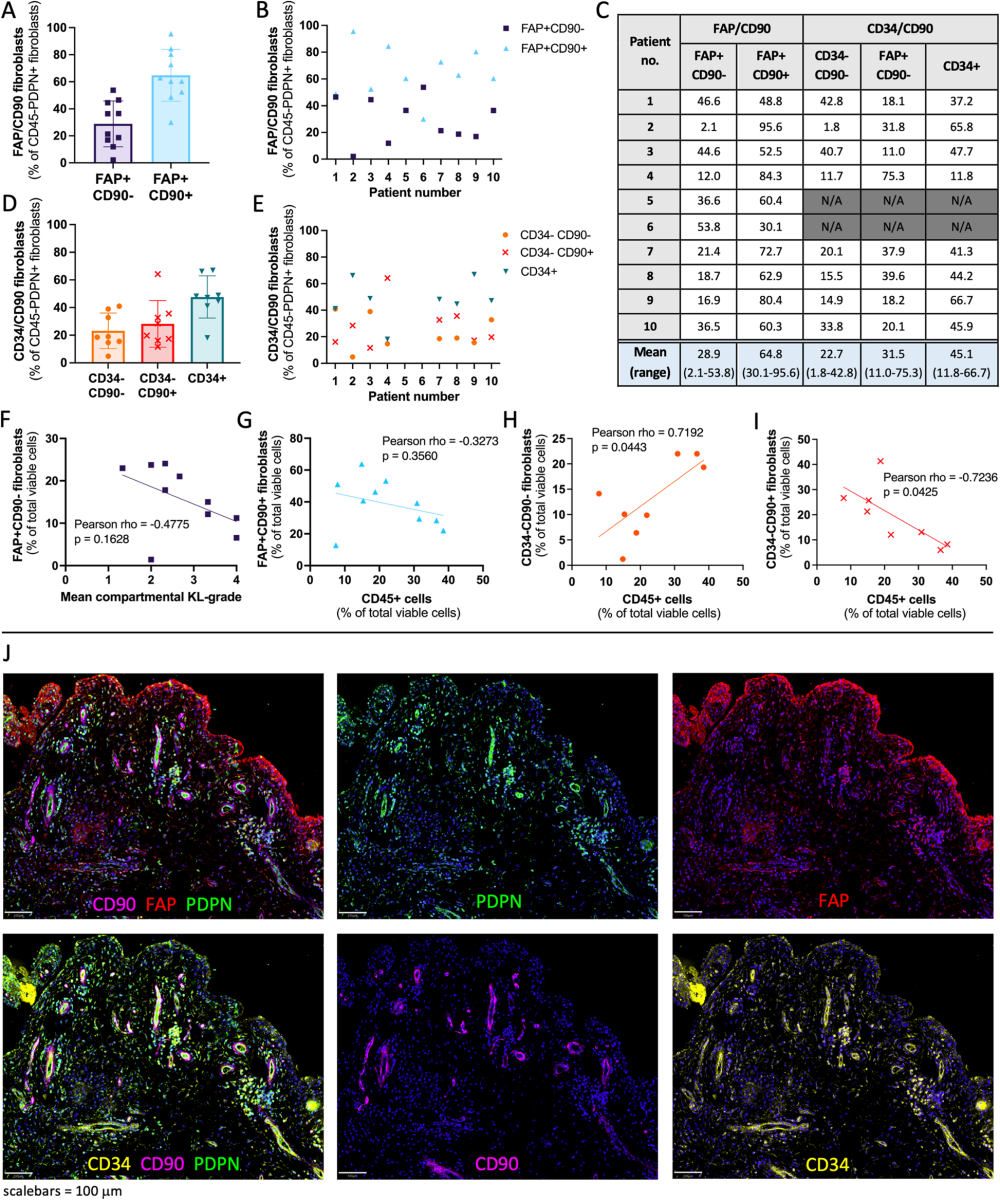
Fibroblast (CD45−PDPN+) subsets in advanced OA synovium. **A**, **B** Relative frequency (%) of fibroblast subsets based on fibroblast activation protein (FAP) and CD90 expression, *n*=10; **C** table with relative frequency (%) of fibroblast subsets based on expression of FAP/CD90 and CD34/CD90. **D**, **E** Relative frequency of fibroblast subsets based on CD34 and CD90 expression, *n*=8; **F**–**I** Relationship between fibroblast subsets as the percentage of all viable cells and mean compartmental Kellgren-Lawrence (KL) grade or immune (CD45+) cells as the percentage of all viable cells. **J** Immunofluorescence staining of fibroblast (activation) markers podoplanin (PDPN, green), FAP (red), CD90 (pink), and CD34 (yellow) in advanced OA synovium; DAPI staining (blue) was used to visualise nuclei

Next, we studied previously identified synovial fibroblast subsets in fibroblasts (CD45−PDPN+ cells). On average 65% of CD45−PDPN+ fibroblasts were FAP+CD90+ fibroblasts (sublining fibroblasts, immune effector phenotype in RA), while 29% of cells were FAP+CD90− (lining fibroblasts, destructive phenotype in RA) (Fig. 5A–C). Most patients (9/10) had more FAP+CD90+ sublining fibroblasts than FAP+CD90− lining fibroblasts (1.05–45.52-fold increase). Exploratory correlative analyses were performed on fibroblast subsets and patient demographics or clinical scores. There seems to be a negative relationship between mean compartmental KL grade and the FAP+CD90− lining fibroblast subset as a percentage of all viable cells, although not significant (Fig. 5F; rho=-0.4775, *p*=0.1628)). No correlation was found between fibroblast subsets and BMI or mean compartmental KL grade (Additional File 10). The relative frequency of FAP+CD90+ sublining fibroblasts as a percentage of all viable cells shows a negative relationship with the relative frequency of CD45+ cells, although not significant (rho= −0.3273, *p*=0.3560). No correlation was found between the relative frequencies of FAP+CD90− fibroblasts and CD45+ cells (Additional File 11).

When studying the expression of CD34/CD90, 23% of fibroblasts were double negative (CD34−CD90), 32% were CD34−CD90+, and 45% were CD34+ (Fig. 5C–E). A significant positive correlation was found between the relative frequencies of CD34−CD90− fibroblasts and CD45+ cells as percentages of all viable cells (Fig. 5H; rho = 0.7192, *p*=0.0443), and a significant negative correlation between the relative frequencies of CD34−CD90+ fibroblasts and CD45+ cells as percentages of all viable cells (F ig. 5I; rho=-0.7236, *p*=0.0425). No correlation was found between the relative frequencies of CD34+ fibroblasts and CD45+ cells (Additional File 11).

Immunofluorescence staining showed that FAP was most strongly expressed in the lining layer, but was also expressed throughout the sublining (Fig. 5J). PDPN was also present within the lining and sublining. Expression of CD34 was mostly found in the sublining layer. In contrast, CD90 was exclusively expressed in the sublining, where it was present around most vessels and in close proximity to CD3+ and CD19+ cells (Additional File 12).

## Discussion

Synovitis is increasingly recognised as an important part of OA pathophysiology, but the immune cells and fibroblast subtypes present, and their potential relationship with clinical scores, are understudied. Therefore, this study set out to characterise the cellular composition of synovium from patients with advanced knee OA requiring total joint replacement and explore the relationship of the identified cell types with patient demographics and clinical scores.

A substantial immune cell population was present in advanced OA synovium in this study, but large differences were found between individuals in both the proportion of immune cells to CD45− cells as well as the relative proportions of lymphocytes and myeloid cells. Macrophages and T cells were confirmed as the first and second most predominant immune cells in advanced OA synovium, but different proportions were found compared to previous studies: 43% macrophages and 34% T cells compared to previous reports of 65% macrophages and 22% T cells [31]. Differences between these studies may be due to different techniques used (immunohistochemistry) and different patient populations (synovial biopsies from OA patients; stage of disease not specified). During exploratory correlative analyses, a significant positive correlation was found between age and macrophages (as percentage of immune cells), and a negative correlation between age and T cells. Macrophages have been reported to be important contributors to chronic inflammation and to the progression of age-related diseases [32]. However, larger studies are needed to investigate this in more detail.

T cells were further characterised with cell surface markers CD8, γδTCR, CD161, and CCR6. CCR6+ T cells were only found in 6 out of 10 patients, who tended to have higher mean compartmental KL grade (higher degree of joint damage) than patients without CCR6+ T cells. CCR6 is a surface receptor for CCL20 and is present on interleukin (IL)-17-producing Th17 T cells and Tregs [33]. Clinical, genetic, *in vitro,* and *in vivo* studies have implicated the IL-17 cytokine family in the pathophysiology of OA [34–39], including evidence that IL-17 can cause OA-like transcriptional changes in chondrocytes and synovial fibroblasts derived from advanced OA patients [40]. CCR6+ T cells have previously been linked to several diseases, including inflammatory bowel disease, systemic lupus erythematosus, psoriasis, and psoriatic arthritis [41–43]. A few studies have also reported findings on CCR6 in OA: increased expression of CCR6 has also been found in OA cartilage compared to healthy donor cartilage [44], and a recent study found that almost half of CD4+ T cells in early-stage OA synovium were positive for CCR6 [45]. To our knowledge, this is the first study to identify CCR6+ T cells in advanced OA synovium. Future studies should investigate whether the presence of CCR6+ T cells might represent a subset of patients with a particularly high degree of joint damage in advanced-stage OA. In order to better understand the potential role of these CCR6+ T cells and its produced cytokine IL-17A in OA pathobiology, it is important that future studies further define these cells as Th17 cells or Tregs, as these two cells types have opposing functions [41].

The proportion of macrophages, the most predominant immune cell type in OA synovium, was highly variable between patients. Half of the macrophages expressed CD206 and only a small proportion expressed CD40. Almost all macrophages positive for CD40 also expressed CD206. This overlap in the expression of these markers, which were previously used as markers for M1 and M2 macrophages, respectively, demonstrates the complexity of macrophage activation and polarisation. Immunofluorescence staining gave further insight in the macrophage subsets based on the expression of CD68, CD163, CD206, and MERTK, which allows the comparison to macrophage subsets used in the first part of the study by Alivernini et al. (2020) based on the expression of CD163, CD206, and MERTK [12]. The expression of CD163 and CD206 largely co-localised; CD163/MERTK, CD206/MERTK, and CD163/CD206/MERTK co-expression was found in some areas, the outside of the lining layer contained mostly CD163+CD206-MERTK- macrophages. This provides early evidence that macrophage subtypes have distinct locations in advanced OA synovium. However, with the identification of 9 different types of synovial tissue macrophages using single-cell RNA sequencing analysis, it is clear that more markers are needed to correctly identify macrophage subsets in synovium. While recent studies have uncovered a lot of information regarding the roles of these different macrophage subsets in RA, more research is needed to understand the potential role of these macrophages in OA. Therefore, future studies require a larger panel of markers to accurately identify synovial tissue macrophages according to the system proposed by Alivernini et al. and use more detailed analyses such as single-cell RNA sequencing to gain insight into the potential function of different synovial tissue macrophage subsets in OA [12, 46].

This study confirmed the expression of fibroblast activation markers CD34, CD90, FAP, and PDPN on CD45− cells in advanced OA synovium, and the presence of previously identified FAP/CD90 and CD34/CD90 fibroblast subsets [21, 22]. For the FAP/CD90 subsets, just over twice as many FAP+CD90+ than FAP+CD90− fibroblasts were found. Interestingly, these ratios are in accordance with ratios previously reported for the RA subset rather than the OA subset [21]. However, it is important to note that these studies used different digestion methods and different analysis methods (flow cytometry vs CyTOF). Future studies should investigate whether the relative proportions of these two FAP/CD90 fibroblasts subsets are an indicator of a phenotype of OA, as it could be hypothesised based on the findings in RA that a high proportion of FAP+CD90+ fibroblasts could result in a more inflammatory disease phenotype, while a high proportion of FAP+CD90− fibroblasts might be linked to a more cartilage-destructive disease phenotype. Next, we investigated the fibroblast subsets based on CD34/CD90 expression [22]. In our study, almost half of fibroblasts were CD34+, after which CD34−CD90+ were most common, closely followed by CD34−CD90− fibroblasts. However, Mizoguichi et al. found that in their OA cohort CD34−CD90− cells were most common, followed by CD34+, and only found few CD34−CD90+ cells. Relative proportions of fibroblast subsets in the advanced OA patients in this study was in accordance with the RA cohort*.* While CD34−CD90+ fibroblasts from RA patients showed a significant positive correlation with the CD45+ population (data was not published for correlations with OA data), in this study CD34−CD90+ fibroblasts showed a significant negative correlation with the CD45+ population. These results suggest that CD34−CD90+ fibroblasts might have a different function in OA compared to RA, in which they are hypothesised to play a role in immune cell recruitment. However, it is important to note that both studies use a small number of patients and use slightly different methods, including different digestion methods and a different selection of fibroblast populations: we studied CD45−PDPN+ cells, while Mizoguichi et al. also excluded red blood cells, endothelial cells, and pericytes from the CD45−PDPN+ population. Immunofluorescence staining confirms that CD90+ fibroblasts were exclusively present in the sublining, while FAP+CD90− fibroblasts were mostly found in the lining layer. CD34+ fibroblasts were mostly found in the sublining, where co-expression with CD90 could be found. The differences between the results of this study and previous studies emphasise the need for research within larger patient cohorts, utilising extensive clinical characteristics, to account for and better understand the observed heterogeneity in patients with advanced stage OA. This also presents an opportunity to investigate whether different ratios of fibroblast subsets in synovium can help identify patient phenotypes.

Limitations of this study include the number of patients analysed, the use of a limited panel of markers to identify cell subsets, and the logistical challenges of using human tissues, including leaving the tissue overnight at 37°C in media to enable the execution of the full flow cytometry protocol in one day. The included patients have a relatively high BMI: only one patient had a normal BMI (18.5–24.9), 2 patients were in the overweight category (BMI 25.0–29.9), 4 patients were in the obese category (30.0–34.9), and 3 patients were in the extremely obese category (BMI 35.0 and over). Although obesity is a known risk factor for OA, this should be considered when interpreting the presented data. The mean compartmental KL grade was used in this study to demonstrate the global disease burden across the joint rather than the classic KL grade system which represents the highest radiographic severity. To facilitate the comparison with the wider OA literature, all results that use the mean compartmental KL grade can also be found in Additional Files 6, 13 (addendum to Figs. 2G and 3F), and 14 (addendum to Fig. 5F and Additional File 10) with the classic KL grade based on medial and lateral compartments only and with the KL grade based on all three compartments. However, more studies are needed to further investigate the use of mean compartmental KL grade, including its validity and reliability when assessing the relationship between radiographic damage of the knee with the synovial cell populations. Synovium was enzymatically digested for flow cytometry analysis, which could have affected cell phenotypes and/or proportions. In addition, this study only looks at synovium from advanced knee OA patients that require a total joint replacement. While this study gives us valuable insights into the immune and fibroblast subsets present in synovium of this subset of advanced OA patients, it is important that future studies compare the cell phenotypes and proportions to those in healthy and early-stage OA synovium to better understand the disease pathophysiology. Finally, (a combination of) single-cell RNA sequencing methods, spectral cytometry, and/or multiplex imaging should be used to further identify the wide range of different cell types and cell subtypes present in OA synovium.

## Conclusions

This study has shown that there are significant differences in the number and subsets of immune cells in OA synovium. Exploratory correlative analyses suggest that these immune cell subsets might correlate with age, clinical scores of disease severity, subtypes of disease, or fibroblast subsets. Interestingly, CCR6+ T cells were only found in a subset of patients, suggesting a potential subtype of disease involving CCR6+ T cells and its produced cytokine IL-17A. Previously identified fibroblast subsets were found, with particularly high proportions of immune effector (FAP+CD90+) fibroblasts. Immunofluorescence revealed that T and B cells, as well as CD90+ fibroblasts, were exclusively present in the sublining layer near vessels, while the lining layer is mainly comprised of FAP+CD90− fibroblasts and CD163+ macrophages. Future studies should further investigate the presence and proportions of different cell types in healthy and OA synovium in combination with patient demographics and clinical scores to investigate whether this could be used to determine disease phenotype.

### Supplementary Information

**Additional file 1.** Overview of antibodies used in each antibody panel of flow cytometry analysis.

**Additional file 2.** Antibodies used for flow cytometry. If concentrations of the antibodywere unknown, recommended concentrations for isotype control antibodies were used. *For the viability stain, no matching isotype control was used. For compensation control, CD4 APC-Cy7 antibody (BioLegend, cat#100526) was used.

**Additional file 3.** Gating strategy for flow cytometry data. (A) Viable cells were selectedby separating cells from debris (SSC-A vs FSC-A), then (B) gating on single cells (SSC-H vs SSC-W), and finally (C) selecting cells negative for the live/dead viability dye (viability dye vs SSC-A). (D) Table of average number of events, cells, single cells, and viable cells for each patient studied.

**Additional file 4.** Overview of primary and secondary antibodies used for immunofluorescence staining.

**Additional file 5.** Overview of cell populations in end-stage OA synovium.

**Additional file 6.** Relationship between T cells and macrophages (as a percentage of all CD45+ cells) and (A) body mass index (BMI), (B) mean compartmental Kellgren-Lawrence (KL)-grade, (C) KL-grade based on the highest radiographic severity in the medial and lateral compartments only (m/l only), and (D) KL-grade based on the highest radiographic severity in the medial, lateral, and patellofemoral compartments (m/l/pf).

**Additional file 7.** Overview of lymphocyte populations in end-stage OA synovium.

**Additional file 8.** Overview of myeloid populations in end-stage OA synovium.

**Additional file 9.** Overview of the expression of fibroblast (activation) markers in CD45- cell population.

**Additional file 10.** Relationship between the relative frequency of fibroblast subsets (as percentage of all viable cells) and body mass index (BMI) (A-E) or mean compartmental Kellgren-Lawrence (KL)-grade (F-J). Fibroblast subsets (CD45-PDPN+) are FAP+CD90- (A, F), FAP+CD90+ (B, G), CD34-CD90- (C, H), CD34-CD90+ (D, I), and CD34+ (E, J).

**Additional file 11.** Relationship between fibroblast subsets (as a percentage of all viablecells) and immune cells (CD45+ cells as a percentage of all viable cells). (A) FAP+CD90- fibroblasts, (B) FAP+CD90+ fibroblasts, (C) CD34-CD90- fibroblasts, (D) CD34-CD90+ fibroblasts, and (E) CD34+ fibroblasts.

**Additional file 12.** Immunofluorescence staining of CD90 (pink), CD146 (orange), and immune cell markers CD3 (cyan), CD19 (yellow), and CD68 (red) in advanced OA synovium; DAPI staining was used to visualise nuclei.

**Additional file 13.** (A) The differences in age, BMI, mean compartmental Kellgren-Lawrence (KL)-grade, KL-grade based on the highest radiographic severity in the medial and lateral compartments only (m/l only), and KL-grade based on the highest radiographic severity in the medial, lateral, and patellofemoral (m/l/pf) compartments between T cell dominant (blue) or macrophage dominant (red) patients. n=10. (Addendum to Figure 2G). (B-D) Relationship between the relative frequency (%) of CCR6+ T cells and age in years mean compartmental Kellgren-Lawrence (KL)-grade (B), KL-grade based on the highest radiographic severity in the medial and lateral compartments only (C), and KL-grade based on the highest radiographic severity in the medial, lateral, and patellofemoral compartments (D). n=10. (Addendum to Figure 3F).

**Additional file 14.** Relationship between the relative frequency of fibroblast subsets (as percentage of all viable cells) and mean compartmental Kellgren-Lawrence (KL)-grade (A-E), KL-grade based on the highest radiographic severity in the medial and lateral compartments only (m/l only) (F-J), and KL-grade based on the highest radiographic severity in the medial, lateral, and patellofemoral (m/l/pf) compartments (K-O). Fibroblast subsets (CD45-PDPN+) are FAP+CD90- (A,F, K), FAP+CD90+ (B, G, L), CD34-CD90- (C, H, M), CD34-CD90+ (D, I, N), and CD34+ (E, J, O). (Addendum to Additional File 10).

## Data Availability

The raw data supporting the conclusion of this article will be made available by the authors, without undue reservation.

## Abbreviations

BMI: Body mass index
CCL: C-C chemokine ligand
CCR: C-C chemokine receptor
CD: Cluster of differentiation
CSB: Cell staining buffer
CyTOF: Cytometry time of flight
FAP: Fibroblast activation protein
FBS: Foetal bovine serum
FSC: Forward scatter
IL: Interleukin
KL grade: Kellgren-Lawrence grade
OA: Osteoarthritis
OKS: Oxford Knee Score
PBS: Phosphate-buffered saline
PDPN: Podoplanin
P/S: Penicillin and streptomycin
R0: RPMI media with 1% penicillin/streptomycin (serum-free)
R10: RPMI media with 10% foetal bovine serum and 1% penicillin/streptomycin
SSC: Side scatter
