# Supplementary material for: Cellular characterisation of advanced osteoarthritis knee synovium
Source: Arthritis Res Ther. 2023 Aug 23;25:154. doi: 10.1186/s13075-023-03110-x (PMC10463598; doi:10.1186/s13075-023-03110-x)
Supplement: Supplementary file 1 — Additional file 1. Overview of antibodies used in each antibody panel of flow cytometry analysis. [file 13075_2023_3110_MOESM1_ESM.pdf]

Additional File 1. Overview of antibodies used in each antibody panel of flow cytometry analysis.

Fluochromes	Detector	Laser	Panel 1: Mixed	Panel 2: T-cells	Panel 3: Granulocytes	Panel 4: Fibroblasts
eFluor 450	Blue	Violet		IL-17AF	IL-17AF	IL-17AF
BV650	Red		CD15	CCR6	CD15	
BV711	Far Red		CD45	CD161	CD45	PDPN
BV786	Infrared		CD34	CD56	CD40	CD34
FITC	Green	Blue	IL-17RC	IL-17A	IL-17A	IL-17A
PE	Yellow		IL-17RA	GDTCR	CD11c	FAP
PE-CF594	Orange		CD19	IL-17F	IL-17F	IL-17F
PerCP/Cy5.5	Red		CD4	CD4	CD117	VCAM
PE-Cy7	Infrared		CD3	CD3	CD68	CD90
APC	Red	Red	CD8	CD8	CD206	CD248
AlexaFluor 700	Far Red		CD14	CD45	CD14	CD45
eFluor 780	Infrared		Viability	Viability	Viability	Viability

White = antibody used during extracellular staining; blue = antibody stained during intracellular staining; black = no antibody used; grey = antibodies that were used for staining but not used for final analysis.
