# Supplementary material for: Cellular characterisation of advanced osteoarthritis knee synovium
Source: Arthritis Res Ther. 2023 Aug 23;25:154. doi: 10.1186/s13075-023-03110-x (PMC10463598; doi:10.1186/s13075-023-03110-x)
Supplement: Supplementary file 2 — Additional file 2. Antibodies used for flow cytometry. If concentrations of the antibodywere unknown, recommended concentrations for isotype control antibodies were used. *For the viability stain, no matching isotype control was used. For compensation control, CD4 APC-Cy7 antibody (BioLegend, cat#100526) was used. [file 13075_2023_3110_MOESM2_ESM.pdf]

Additional File 2. Antibodies used for flow cytometry. If concentrations of the antibody were unknown, recommended concentrations for isotype control antibodies were used. *For the viability stain, no matching isotype control was used. For compensation control, CD4 APC-Cy7 antibody (BioLegend, cat# 100526) was used.

**Antibodies**							**Matching isotype controls**					
**Target**	**Company**	**Fluo-chrome**	**Isotype**	**Clone**	**Cat number**	**Amount used (µL/µg)**	**Company**	**Fluo-chrome**	**Isotype**	**Clone**	**Cat number**	**Amount used (µL/µg)**
**CD106 (VCAM)**	BD	PerCP/Cy5.5	Mouse IgG1, kappa	51-10C9	563525	5 / 0.5	BD	PerCP/Cy5.5	Mouse IgG1, kappa	MOPC-21	552834	10 / 0.5
**CD117**	BD	PerCP/Cy5.5	Mouse IgG1, kappa	YB5.B8	562094	5 / 0.125	BD	PerCP/Cy5.5	Mouse IgG1, kappa	MOPC-21	552834	2.5 / 0.125
**CD11c**	Miltenyi Biotech	PE	Mouse IgG2b, kappa	MJ4-27G12	130-099-212	2 / unknown	Miltenyi Biotech	PE	Mouse IgG2b, kappa	IS6-11E5.11	130-098-875	2 / unknown
**CD14**	BioLegend	AlexaFluor 700	Mouse IgG2a, kappa	M5E2	301822	4 / 2	BioLegend	AlexaFluor 700	Mouse IgG2a, kappa	MOPC-173	400247	4 / 2
**CD15**	BD	BV650	Mouse IgM, kappa	HI98	564232	5 / 0.125	BD	BV650	Mouse IgM, kappa	G155-228	564367	0.625 / 0.125
**CD161**	BD	BV711	Mouse C3H (C3H/Bi) IgG1, kappa	CX12	563865	5 / 0.5	BD	BV711	Mouse BALB/c IgG1, kappa	X40	563044	2.5 / 0.5
**CD19**	BD	PE-CF594	Mouse IgG1, kappa	HIB19	562321	5 / 0.25	BD	PE-CF594	Mouse IgG1, kappa	X40	562292	1.25 / 0.25
**CD196 (CCR6)**	BD	BV650	Mouse IgG1, kappa	11A9	563922	5 / 0.25	BD	BV650	Mouse IgG1, kappa	X40	563231	1.25 / 0.25
**CD206**	BioLegend	APC	Mouse IgG1, kappa	15-2	321109	5 / 1	BioLegend	APC	Mouse IgG1, kappa	MOPC-21	400119	5 / 1
**CD248**	BD	AF647	Mouse IgG1, kappa	B1/35	564994	5 / 0.5	BD	AF647	Mouse IgG1, kappa	MOPC-21	557714	5 / 0.5
**CD3**	BioLegend	PE-Cy7	Mouse IgG1, kappa	SK7	344816	5 / 2	BioLegend	PE-Cy7	Mouse IgG1, kappa	MOPC-21	400125	5 / 0.5
**CD34**	BioLegend	BV785	Mouse IgG2a, kappa	561	343625	5 / 0.25	BioLegend	BV785	Mouse IgG2a, kappa	MOPC-173	400273	2.5 / 0.25
**CD4**	BioLegend	PerCP/Cy5.5	Mouse IgG1, kappa	RPA-T4	300530	5 / 0.5	BioLegend	PerCP/Cy5.5	Mouse IgG1, kappa	MOPC-21	400149	2.5 / 0.5

**CD40**	BioLegend	BV785	Mouse IgG1, kappa	5C3	334339	5 / 0.25	BioLegend	BV785	Mouse IgG1, kappa	MOPC-21	400169	2.5 / 0.25
**CD45**	BioLegend	AlexaFluor 700	Mouse IgG1, kappa	HI30	304023	2 / 1	BioLegend	AlexaFluor 700	Mouse IgG1, kappa	MOPC-21	400143	2 / 1
**CD45**	BioLegend	BV711	Mouse IgG1, kappa	HI30	304050	2 / 0.1	BioLegend	BV711	Mouse IgG1, kappa	MOPC-21	400167	1 / 0.1
**CD56**	BD	BV786	Mouse BALB/c IgG2b, kappa	NCAM1 6.2	564058	5 / 0.25	BD	BV786	Mouse C.SW IgG2b	27-35	564090	1.25 / 0.25
**CD68**	BioLegend	PE-Cy7	Mouse IgG2b, kappa	Y1/82A	333815	5 / 0.06	BioLegend	PE-Cy7	Mouse IgG2b, kappa	MPC-11	400325	0.3 / 0.06
**CD8**	BD	APC	Mouse IgG1, kappa	RPA-T8	561952/555369	8 / 0.096	BD	APC	Mouse IgG1, kappa	MOPC-21	560167	0.48 / 0.096
**CD90**	BioLegend	PE-Cy7	Mouse IgG1, kappa	5E10	328123	5 / 0.5	BioLegend	PE-Cy7	Mouse IgG1, kappa	MOPC-21	400125	5 / 0.5
**FAP**	R&D	PE	Mouse IgG1, kappa	427819	FAB3715P-100	10 / unknown	R&D	PE	Mouse IgG1, kappa	11711	IC002P	10 / unknown
**GDTCR**	BioLegend	PE	Mouse IgG1, kappa	B1	331209	5 / 2	BioLegend	PE	Mouse IgG1, kappa	MOPC-21	400113	10 / 2
**IL-17A**	eBioScience	FITC	Mouse IgG1, kappa	eBio64DEC17	11-7179-42	5 / 0.125	eBioScience	FITC	Mouse IgG1, kappa	P3.6.2.8.1	11-4714-81	0.25 / 0.125
**IL-17AF**	eBioScience	eF450	Mouse IgG1, kappa	20LJS09	48-9179-42	5 / 0.5	eBioScience	eFluor 450	Mouse IgG1, kappa	P3.6.2.8.1	48-4714-82	2.5 / 0.5
**IL-17F**	BD	PE-CF594	Mouse IgG1, kappa	O33-782	564263	5 / 0.25	BD	PE-CF594	Mouse IgG1, kappa	X40	562292	1.25 / 0.25
**IL-17RA**	Miltenyi Biotech	PE	Recombinant human IgG1	REA290	130-104-768	9 / unknown	Miltenyi Biotech	PE	Recombinant human IgG1	REA293	130-113-438	2 / unknown
**IL-17RC**	Miltenyi Biotech	FITC	Recombinant human IgG1	REA571	130-109-196	9 / unknown	Miltenyi Biotech	FITC	Recombinant human IgG1	REA293	130-104-610	2 / unknown
**PDPN**	BD	BV711	Mouse BALB/c IgG1, kappa	LpMab-17	747632	5 / 1	BD	BV711	Mouse BALB/c IgG1, kappa	X40	563044	5 / 1
**Viability***	eBioScience	BV780	-	-	65-0865-14	0.4	-	-	-	-	-	-
