# Supplementary material for: Cellular characterisation of advanced osteoarthritis knee synovium
Source: Arthritis Res Ther. 2023 Aug 23;25:154. doi: 10.1186/s13075-023-03110-x (PMC10463598; doi:10.1186/s13075-023-03110-x)
Supplement: Supplementary file 3 — Additional file 3. Gating strategy for flow cytometry data. (A) Viable cells were selectedby separating cells from debris (SSC-A vs FSC-A), then (B) gating on single cells (SSC-H vs SSC-W), and finally (C) selecting cells negative for the live/dead viability dye (viability dye vs SSC-A). (D) Table of average number of events, cells, single cells, and viable cells for each patient studied. [file 13075_2023_3110_MOESM3_ESM.pdf]

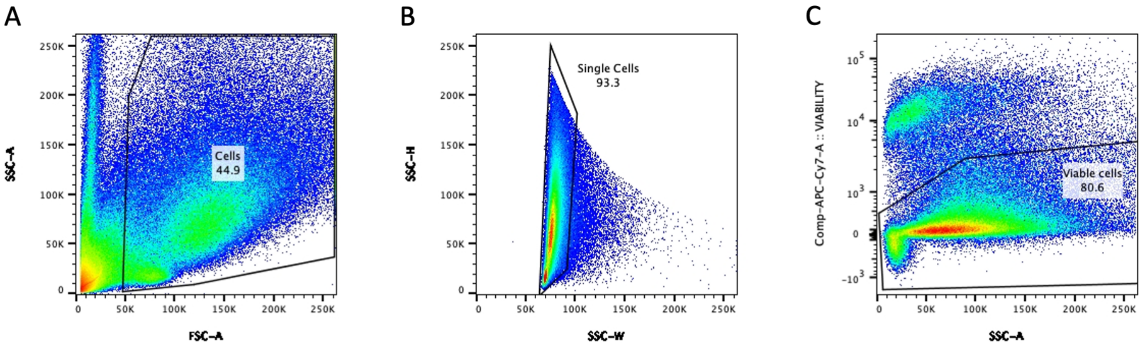

D

Patient no.	No. events (mean)	Cells (% of events)	Single cells (% of cells)	Viable cells (% of single cells)
1	98,284	41.5	92.3	66.2
2	83,567	43.4	92.1	80.1
3	411,036	60.3	92.9	83.2
4	158,841	46.2	93.6	82.4
5	296,111	59.6	92.0	68.4
6	175,666	9.1	89.7	82.5
7	242,033	62.3	92.3	80.0
8	341,518	45.4	94.2	78.2
9	148,931	46.2	95.3	77.9
10	537,429	67.1	95.1	85.9

Additional File 3. Gating strategy for flow cytometry data. (A) Viable cells were selected by separating cells from debris (SSC-A vs FSC-A), then (B) gating on single cells (SSC-H vs SSC-W), and finally (C) selecting cells negative for the live/dead viability dye (viability dye vs SSC-A). (D) Table of average number of events, cells, single cells, and viable cells for each patient studied.
