# Supplementary material for: Cellular characterisation of advanced osteoarthritis knee synovium
Source: Arthritis Res Ther. 2023 Aug 23;25:154. doi: 10.1186/s13075-023-03110-x (PMC10463598; doi:10.1186/s13075-023-03110-x)
Supplement: Supplementary file 4 — Additional file 4. Overview of primary and secondary antibodies used for immunofluorescence staining. [file 13075_2023_3110_MOESM4_ESM.pdf]

Additional File 4. Overview of primary and secondary antibodies used for immunofluorescence staining.

Primary antibodies						
**Target**	**Isotype**	**Clone**	**Conjugation**	**Supplier**	**Ref no.**	**Concentration**
**CD146**	Rabbit IgG	EPR3208	488	Abcam	ab75769	10 mg/ml
**CD163**	Mouse IgG1	EDHu-1	647	Novus	NB110-40686	5 mg/ml
**CD19**	Rabbit IgG	EPR5906	647	Abcam	ab196515	10 mg/ml
**CD206**	Mouse IgG2a	C-10	546	SCBT	sc-376232	10 mg/ml
**CD3**	Rabbit IgG	SP162	555	Abcam	ab245731	10 mg/ml
**CD34**	Rabbit IgG	EP373Y	488	Abcam	ab195013	10 mg/ml
**CD45**	Mouse IgG2a	2D1	647	Biolegend	368538	5 mg/ml
**CD68**	Rabbit IgG	EPR20545	555	Abcam	ab280860	5 mg/ml
**CD8**	Mouse IgG1	C8/144B	647	Biolegend	372906	10 mg/ml
**CD90**	Rabbit IgG	EPR3132	555	Abcam	ab181469	10 mg/ml
**FAP**	Polyclonal sheep IgG	Polyclonal	Unconjugated	R&D	AF3715	10 mg/ml
**MERTK**	Rabbit IgG	Y323	450	Abcam	ab271851	10 mg/ml
**PDPN**	Rat IgG2a	NZ-1.3	Unconjugated	Thermo	14-9381-82	5 mg/ml
**Secondary antibodies**						
**Target**	**Isotype**	**Clone**	**Conjugation**	**Supplier**	**Ref no.**	**Concentration**
Donkey anti-rabbit	IgG	Polyclonal	488	Thermo	A21206	1:500 (2 µg/ml)
Donkey anti-rat	IgG	Polyclonal	555	Abcam	ab150254	1:500 (2 µg/ml)
Donkey anti-sheep	IgG	Polyclonal	647	Jackson ImmunoResearch	713-606-147	1:500 (2 µg/ml)
