# Supplementary material for: Cellular characterisation of advanced osteoarthritis knee synovium
Source: Arthritis Res Ther. 2023 Aug 23;25:154. doi: 10.1186/s13075-023-03110-x (PMC10463598; doi:10.1186/s13075-023-03110-x)
Supplement: Supplementary file 5 — Additional file 5. Overview of cell populations in end-stage OA synovium. [file 13075_2023_3110_MOESM5_ESM.pdf]

Additional File 5. Overview of cell populations in end-stage OA synovium.

Patient no.	Lymphocytes (%)	Myeloid cells (%)	CD45+ total (%)	CD45- (%)
**1**	21.6	16.2	37.8	58.0
**2**	7.7	8.2	15.9	82.7
**3**	8.2	27.8	36.0	61.9
**4**	8.1	8.5	16.6	81.6
**5**	11.4	19.2	30.6	67.7
**6**	7.2	4.3	11.5	87.6
**7**	1.1	6.6	7.7	90.7
**8**	9.1	5.9	15.1	83.4
**9**	9.2	13.1	22.3	76.7
**10**	8.2	21.7	29.9	69.1
**Mean (range)**	9.2 (1.1-21.6)	13.2 (4.3-27.8)	22.3 (7.7-37.8)	75.9 (58.0-90.7)

Relative frequencies (%) of CD45 subsets (as explained in Figure 1) as a percentage of viable cells in end-stage OA synovium.
