# Supplementary material for: Cellular characterisation of advanced osteoarthritis knee synovium
Source: Arthritis Res Ther. 2023 Aug 23;25:154. doi: 10.1186/s13075-023-03110-x (PMC10463598; doi:10.1186/s13075-023-03110-x)
Supplement: Supplementary file 6 — Additional file 6. Relationship between T cells and macrophages (as a percentage of all CD45+ cells) and (A) body mass index (BMI), (B) mean compartmental Kellgren-Lawrence (KL)-grade, (C) KL-grade based on the highest radiographic severity in the medial and lateral compartments only (m/l only), and (D) KL-grade based on the highest radiographic severity in the medial, lateral, and patellofemoral compartments (m/l/pf). [file 13075_2023_3110_MOESM6_ESM.pdf]

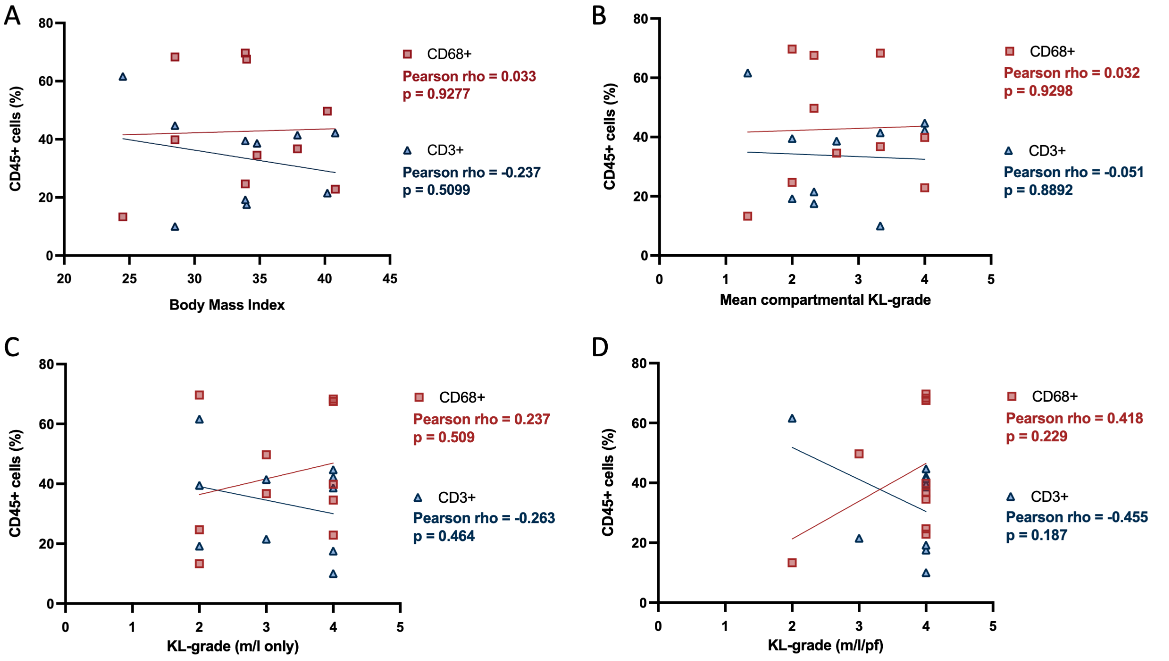

Additional File 6. Relationship between T cells and macrophages (as a percentage of all CD45+ cells) and (A) body mass index (BMI), (B) mean compartmental Kellgren-Lawrence (KL)-grade, (C) KL-grade based on the highest radiographic severity in the medial and lateral compartments only (m/l only), and (D) KL-grade based on the highest radiographic severity in the medial, lateral, and patellofemoral compartments (m/l/pf).
