# Supplementary material for: Cellular characterisation of advanced osteoarthritis knee synovium
Source: Arthritis Res Ther. 2023 Aug 23;25:154. doi: 10.1186/s13075-023-03110-x (PMC10463598; doi:10.1186/s13075-023-03110-x)
Supplement: Supplementary file 7 — Additional file 7. Overview of lymphocyte populations in end-stage OA synovium. [file 13075_2023_3110_MOESM7_ESM.pdf]

Additional File 7. Overview of lymphocyte populations in end-stage OA synovium.

Patient no.	As % of lymphocytes		As % of CD3 cells			
	**CD3+**	**CD19+**	**CCR6+**	**CD8+**	**CD161+**	**GDTCR+**
**1**	81.2	6.4	0.1	25.9	4.2	1.5
**2**	91.0	1.6	0.6	37.9	1.1	1.1
**3**	77.3	15.5	29.2	34.1	0.5	2.0
**4**	92.1	0.3	36.2	40.2	0.4	3.4
**5**	56.6	31.1	0.0	18.9	0.1	N/A
**6**	95.4	0.7	0.2	11.7	0.0	N/A
**7**	85.8	3.3	35.3	39.7	1.1	1.8
**8**	74.6	20.0	36.2	42.4	0.1	2.3
**9**	87.5	4.9	19.2	21.4	0.2	0.2
**10**	76.6	10.1	26.9	28.3	0.1	0.7
**Mean (range)**	81.9 (56.6-95.4)	9.4 (0.3-31.1)	18.4 (0.02-36.2)	30.1 (11.7-42.4)	0.8 (0.0-4.2)	1.6 (0.2-3.4)

Overview of the relative frequencies (%) of CD3+ and CD19+ cells as a percentage of lymphocyte population, and overview of CD3+ subsets.
