# Supplementary material for: Cellular characterisation of advanced osteoarthritis knee synovium
Source: Arthritis Res Ther. 2023 Aug 23;25:154. doi: 10.1186/s13075-023-03110-x (PMC10463598; doi:10.1186/s13075-023-03110-x)
Supplement: Supplementary file 8 — Additional file 8. Overview of myeloid populations in end-stage OA synovium. [file 13075_2023_3110_MOESM8_ESM.pdf]

Additional File 8. Overview of myeloid populations in end-stage OA synovium.

Patient no.	As % of myeloid cells						As % of CD68+ myeloid cells		
	CD11c+	CD14+	CD15+	CD40+	CD68+	CD206+	CD40+	CD206+	CD40+ CD206+
1	11.8	25.4	5.8	27.1	67.5	55.9	24.1	47.4	21.1
2	14.8	26.1	1.3	7.8	48.5	59.9	7.4	34.2	6.4
3	3.3	29.1	0.6	3.9	89.8	70.9	3.8	71.2	3.0
4	11.6	36.8	0.5	14.0	42.6	72.8	14.6	58.9	13.0
5	7.3	24.1	0.7	6.1	77.5	40.0	5.1	32.8	4.7
6	8.8	14.5	5.0	12.7	43.5	64.0	17.1	48.8	4.1
7	11.4	37.6	0.5	6.1	80.7	56.5	5.5	51.2	2.8
8	12.8	28.2	0.8	12.5	89.7	54.7	12.7	57.5	10.7
9	7.2	34.0	0.2	21.7	78.6	49.1	20.5	45.8	18.6
10	6.1	30.7	0.5	9.8	93.5	36.5	9.5	37.3	9.1
**Mean (range)**	9.5 (3.3-14.8)	28.7 (14.5-37.6)	1.6 (0.2-5.8)	12.2 (3.9-27.1)	71.2 (42.6-93.5)	56.0 (36.5-72.8)	12.0 (3.8-24.1)	48.5 (32.8-71.2)	9.4 (2.8-21.1)

Overview of the relative frequencies (%) of CD11c+, CD14+, CD15+, CD40+, CD68+, and CD206+ cells as a percentage of myeloid cells, and overview of CD68+ subsets.
