# Supplementary material for: Cellular characterisation of advanced osteoarthritis knee synovium
Source: Arthritis Res Ther. 2023 Aug 23;25:154. doi: 10.1186/s13075-023-03110-x (PMC10463598; doi:10.1186/s13075-023-03110-x)
Supplement: Supplementary file 9 — Additional file 9. Overview of the expression of fibroblast (activation) markers in CD45- cell population. [file 13075_2023_3110_MOESM9_ESM.pdf]

Additional File 9. Overview of the expression of fibroblast (activation) markers in CD45- cell population.

Patient no.	CD34+		CD90+		FAP+		PDPN+	
	AB	ISO	AB	ISO	AB	ISO	AB	ISO
1	41.1	0.12	45.4	0.072	77.7	0.77	77.9	0.33
2	66.1	0.049	85.8	0.0	82.5	1.37	80.7	0.73
3	48.8	0.32	52.6	0.14	91.2	3.27	87.3	3.08
4	18.1	0.36	76.5	0.045	75.9	1.56	67.3	2.76
5	N/A	N/A	53.0	0.069	81.1	0.43	72.0	0.26
6	N/A	N/A	20.4	0.71	44.7	2.58	48.8	1.51
7	48.2	0.3	63.4	0.076	77.5	1.91	77.4	2.05
8	44.7	0.46	60.1	0.15	70.7	1.54	77.7	0.81
9	66.9	0.22	75.4	0.2	88.8	1.17	86.3	1.28
10	47.2	0.11	61.9	0.061	93.3	1.33	94.2	0.47
**Mean (range)**	47.6 (18.1-66.9)	0.2 (0.0-0.5)	59.5 (20.4-85.8)	0.2 (0.0-0.7)	78.3 (44.7-93.3)	1.6 (0.4-3.3)	77.0 (48.8-94.2)	1.3 (0.3-3.1)

Table presenting the relative frequencies (%) of positive populations and isotype controls of CD34, CD90, FAP, and PDPN.
