# Supplementary material for: Cellular characterisation of advanced osteoarthritis knee synovium
Source: Arthritis Res Ther. 2023 Aug 23;25:154. doi: 10.1186/s13075-023-03110-x (PMC10463598; doi:10.1186/s13075-023-03110-x)
Supplement: Supplementary file 10 — Additional file 10. Relationship between the relative frequency of fibroblast subsets (as percentage of all viable cells) and body mass index (BMI) (A-E) or mean compartmental Kellgren-Lawrence (KL)-grade (F-J). Fibroblast subsets (CD45-PDPN+) are FAP+CD90- (A, F), FAP+CD90+ (B, G), CD34-CD90- (C, H), CD34-CD90+ (D, I), and CD34+ (E, J). [file 13075_2023_3110_MOESM10_ESM.pdf]

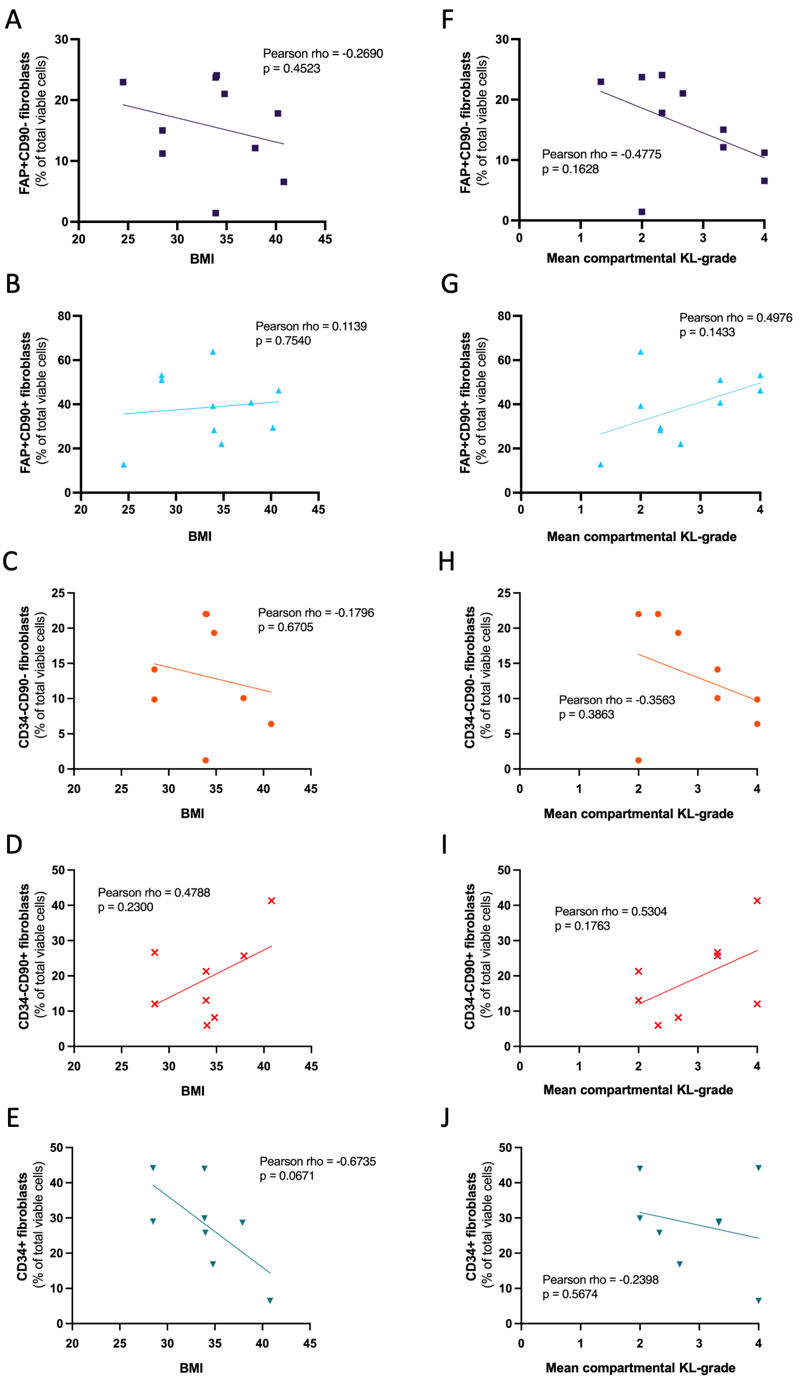

Additional File 10. Relationship between the relative frequency of fibroblast subsets (as percentage of all viable cells) and body mass index (BMI) (A-E) or mean compartmental Kellgren-Lawrence (KL)-grade (F-J). Fibroblast subsets (CD45-PDPN+) are FAP+CD90- (A, F), FAP+CD90+ (B, G), CD34-CD90- (C, H), CD34-CD90+ (D, I), and CD34+ (E, J).
