# Supplementary material for: Cellular characterisation of advanced osteoarthritis knee synovium
Source: Arthritis Res Ther. 2023 Aug 23;25:154. doi: 10.1186/s13075-023-03110-x (PMC10463598; doi:10.1186/s13075-023-03110-x)
Supplement: Supplementary file 11 — Additional file 11. Relationship between fibroblast subsets (as a percentage of all viablecells) and immune cells (CD45+ cells as a percentage of all viable cells). (A) FAP+CD90- fibroblasts, (B) FAP+CD90+ fibroblasts, (C) CD34-CD90- fibroblasts, (D) CD34-CD90+ fibroblasts, and (E) CD34+ fibroblasts. [file 13075_2023_3110_MOESM11_ESM.pdf]

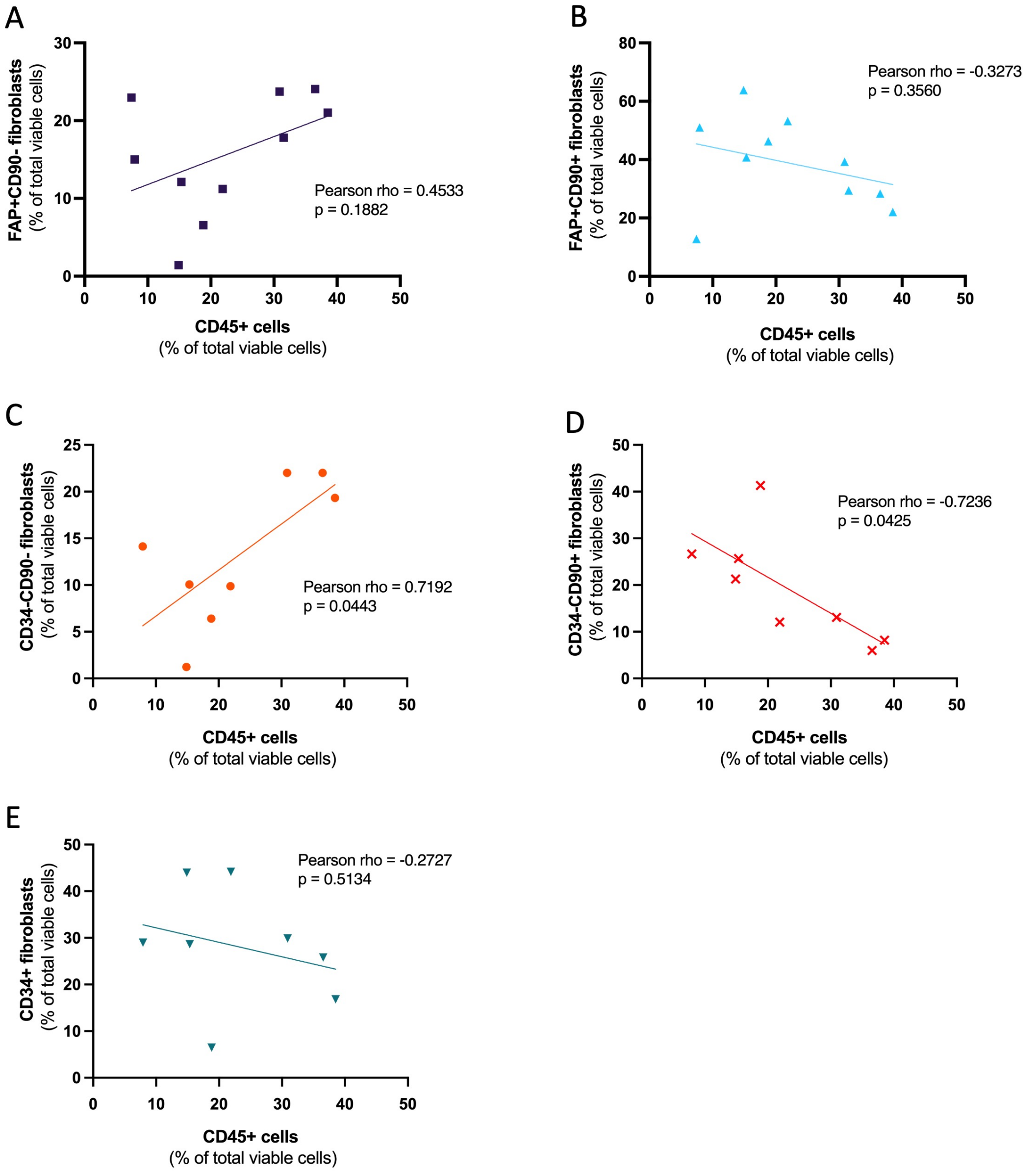

Additional File 11. Relationship between fibroblast subsets (as a percentage of all viable cells) and immune cells (CD45+ cells as a percentage of all viable cells). (A) FAP+CD90- fibroblasts, (B) FAP+CD90+ fibroblasts, (C) CD34-CD90- fibroblasts, (D) CD34-CD90+ fibroblasts, and (E) CD34+ fibroblasts.
