# Supplementary material for: Cellular characterisation of advanced osteoarthritis knee synovium
Source: Arthritis Res Ther. 2023 Aug 23;25:154. doi: 10.1186/s13075-023-03110-x (PMC10463598; doi:10.1186/s13075-023-03110-x)
Supplement: Supplementary file 12 — Additional file 12. Immunofluorescence staining of CD90 (pink), CD146 (orange), and immune cell markers CD3 (cyan), CD19 (yellow), and CD68 (red) in advanced OA synovium; DAPI staining was used to visualise nuclei. [file 13075_2023_3110_MOESM12_ESM.pdf]

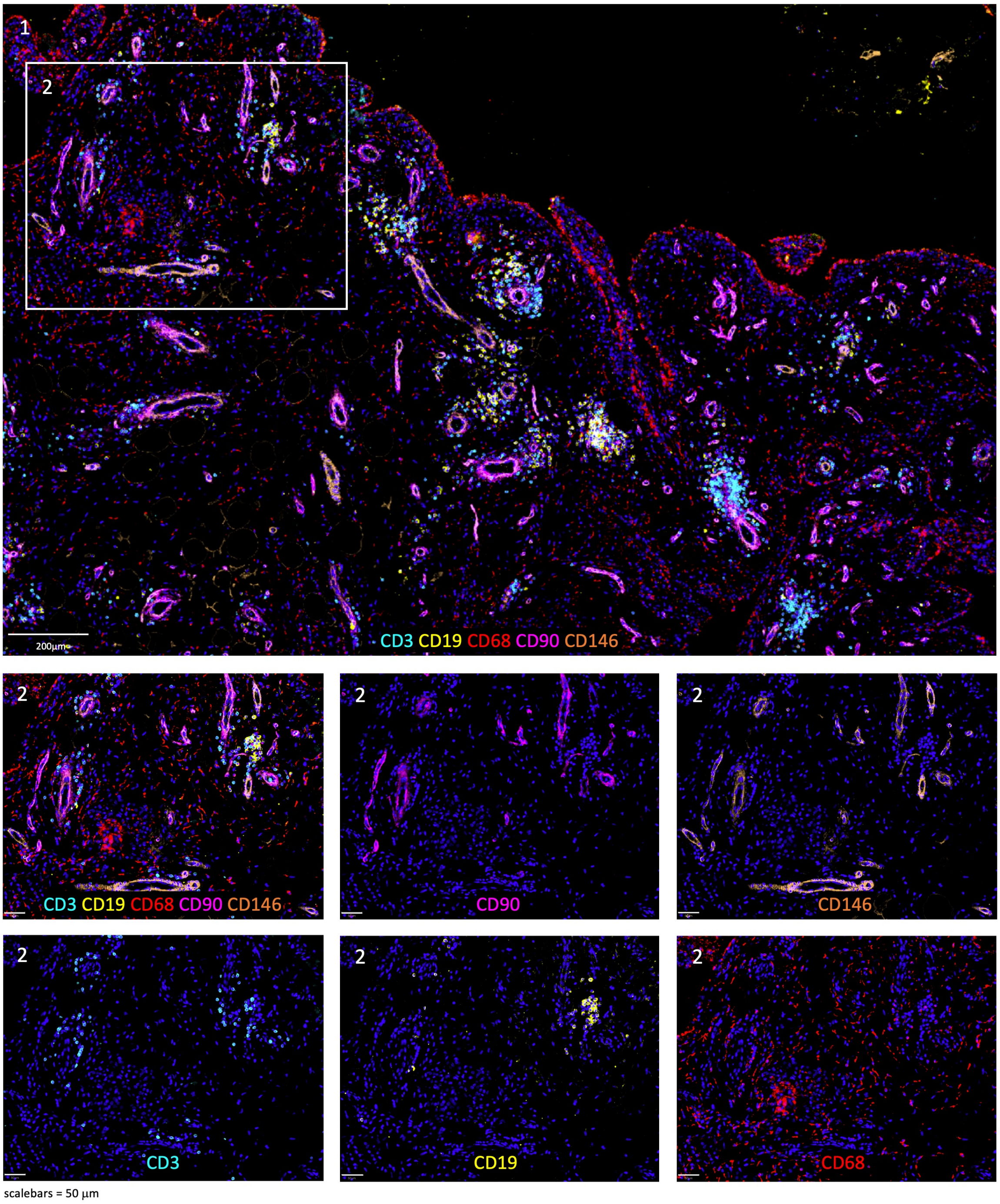

Additional File 12. Immunofluorescence staining of CD90 (pink), CD146 (orange), and immune cell markers CD3 (cyan), CD19 (yellow), and CD68 (red) in advanced OA synovium; DAPI staining was used to visualise nuclei.
