# Supplementary material for: Cellular characterisation of advanced osteoarthritis knee synovium
Source: Arthritis Res Ther. 2023 Aug 23;25:154. doi: 10.1186/s13075-023-03110-x (PMC10463598; doi:10.1186/s13075-023-03110-x)
Supplement: Supplementary file 13 — Additional file 13. (A) The differences in age, BMI, mean compartmental Kellgren-Lawrence (KL)-grade, KL-grade based on the highest radiographic severity in the medial and lateral compartments only (m/l only), and KL-grade based on the highest radiographic severity in the medial, lateral, and patellofemoral (m/l/pf) compartments between T cell dominant (blue) or macrophage dominant (red) patients. n=10. (Addendum to Figure 2G). (B-D) Relationship between the relative frequency (%) of CCR6+ T cells and age in years mean compartmental Kellgren-Lawrence (KL)-grade (B), KL-grade based on the highest radiographic severity in the medial and lateral compartments only (C), and KL-grade based on the highest radiographic severity in the medial, lateral, and patellofemoral compartments (D). n=10. (Addendum to Figure 3F). [file 13075_2023_3110_MOESM13_ESM.pdf]

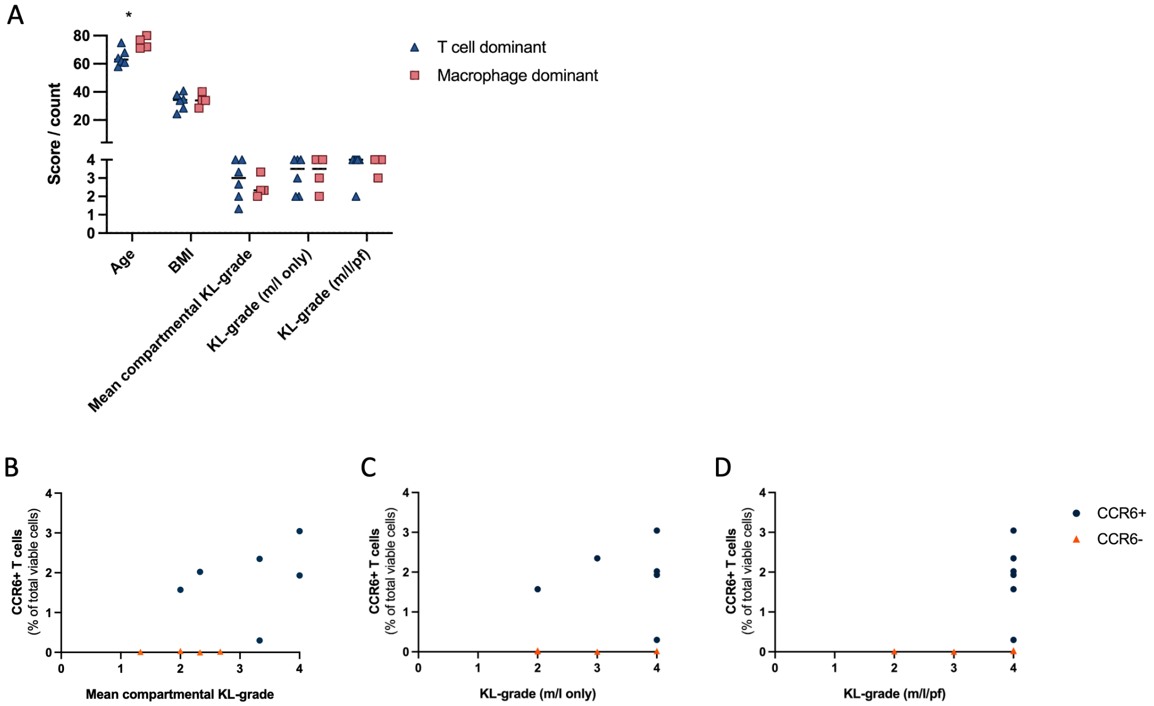

Additional File 13. (A) The differences in age, BMI, mean compartmental Kellgren-Lawrence (KL)-grade, KL-grade based on the highest radiographic severity in the medial and lateral compartments only (m/l only), and KL-grade based on the highest radiographic severity in the medial, lateral, and patellofemoral (m/l/pf) compartments between T cell dominant (blue) or macrophage dominant (red) patients. n=10. (Addendum to Figure 2G). (B-D) Relationship between the relative frequency (%) of CCR6+ T cells and age in years mean compartmental Kellgren-Lawrence (KL)-grade (B), KL-grade based on the highest radiographic severity in the medial and lateral compartments only (C), and KL-grade based on the highest radiographic severity in the medial, lateral, and patellofemoral compartments (D). n=10. (Addendum to Figure 3F).
