# Supplementary material for: Cellular characterisation of advanced osteoarthritis knee synovium
Source: Arthritis Res Ther. 2023 Aug 23;25:154. doi: 10.1186/s13075-023-03110-x (PMC10463598; doi:10.1186/s13075-023-03110-x)
Supplement: Supplementary file 14 — Additional file 14. Relationship between the relative frequency of fibroblast subsets (as percentage of all viable cells) and mean compartmental Kellgren-Lawrence (KL)-grade (A-E), KL-grade based on the highest radiographic severity in the medial and lateral compartments only (m/l only) (F-J), and KL-grade based on the highest radiographic severity in the medial, lateral, and patellofemoral (m/l/pf) compartments (K-O). Fibroblast subsets (CD45-PDPN+) are FAP+CD90- (A,F, K), FAP+CD90+ (B, G, L), CD34-CD90- (C, H, M), CD34-CD90+ (D, I, N), and CD34+ (E, J, O). (Addendum to Additional File 10). [file 13075_2023_3110_MOESM14_ESM.pdf]

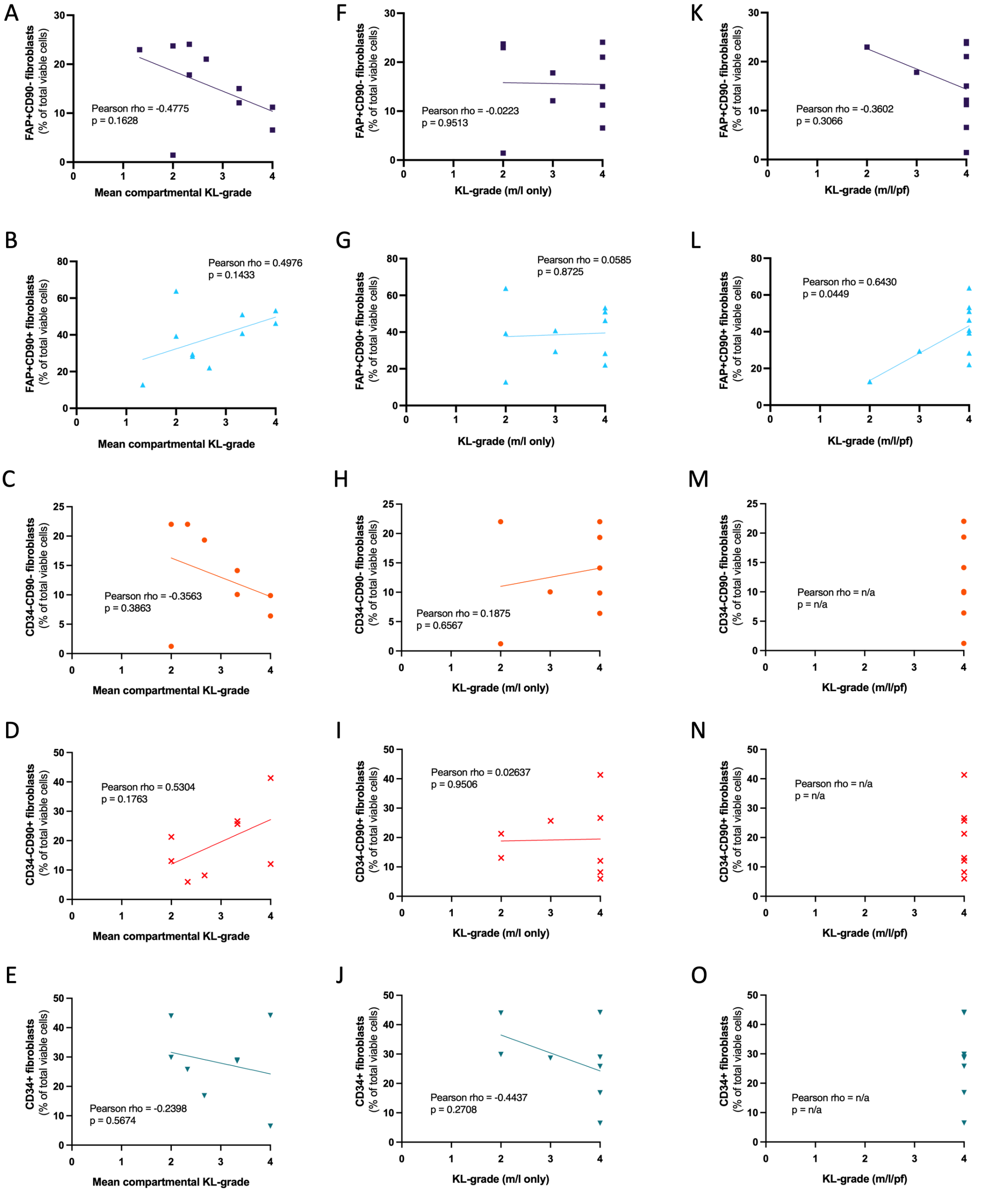

Additional File 14. Relationship between the relative frequency of fibroblast subsets (as percentage of all viable cells) and mean compartmental Kellgren-Lawrence (KL)-grade (A-E), KL-grade based on the highest radiographic severity in the medial and lateral compartments only (m/l only) (F-J), and KL-grade based on the highest radiographic severity in the medial, lateral, and patellofemoral (m/l/pf) compartments (K-O). Fibroblast subsets (CD45-PDPN+) are FAP+CD90- (A, F, K), FAP+CD90+ (B, G, L), CD34-CD90- (C, H, M), CD34-CD90+ (D, I, N), and CD34+ (E, J, O). (Addendum to Additional File 10).
